# Estimating the causal effect of redlining on present-day air pollution

**DOI:** 10.1093/biomtc/ujaf173

**Published:** 2026-01-15

**Authors:** Xiaodan Zhou, Shu Yang, Brian J Reich

**Affiliations:** Department of Statistics, North Carolina State University, Raleigh, NC 27695, United States; Department of Statistics, North Carolina State University, Raleigh, NC 27695, United States; Department of Statistics, North Carolina State University, Raleigh, NC 27695, United States

**Keywords:** air pollution exposure, latent factor model, proxy variable, redlining policy, spatial causal model

## Abstract

Recent studies have shown associations between redlining policies (1935–1974) and present-day fine particulate matter (PM$_{2.5}$) and nitrogen dioxide (NO$_2$) air pollution concentrations. In this paper, we move beyond associations and investigate the causal effects of redlining using spatial causal inference. Redlining policies were enacted in the 1930s, so there is very limited documentation of pre-treatment covariates. Consequently, traditional methods failed to sufficiently account for unmeasured confounders, potentially biasing causal interpretations. By integrating historical redlining data with 2010 PM$_{2.5}$ and NO$_2$ concentrations, our study seeks to estimate the long-term causal impact. Our study addresses challenges with a novel spatial and non-spatial latent factor framework, using the unemployment rate, house rent and percentage of Black population in 1940 US Census as proxies to reconstruct pre-treatment latent socio-economic status. We establish identification of a causal effect under broad assumptions, and use Bayesian Markov Chain Monte Carlo to quantify uncertainty. Our causal analysis provides evidence that historically redlined neighborhoods are exposed to notably higher NO$_2$ concentration. In contrast, the disparities in PM$_{2.5}$ between these neighborhoods are less pronounced. Among the cities analyzed, Los Angeles, CA, and Atlanta, GA, demonstrate the most significant effects for both NO$_2$ and PM$_{2.5}$.

## 1 INTRODUCTION

### 1.1 Redlining policy

The redlining policy, initiated in 1935 by the Federal Home Loan Bank Board, mandated the Home Owners’ Loan Corporation (HOLC) to create “residential security maps.” These maps classified residential regions with grades reflecting investment security: “A” for Desirable, “B” for Still Desirable, “C” for Declining, and “D” for Redlined. This grading system, operational until the 1974, directly influenced lending decisions. Regions graded “A” were considered minimal risk by banks and mortgage lenders for loans and safe investments, while those labeled “D” were deemed hazardous.

Some studies have investigated the financial inequalities stemming from the redlining policy, with a emphasis on causal analysis and addressing potential confounders. Aaronson et al. (2021) employed a boundary-design approach to mitigate these confounders. Their analysis concentrated on areas adjacent to redlining boundaries (D), comparing “treated” and “controlled” boundaries using propensity score weighting. They discovered that regions assigned to be redlined experienced deteriorating housing market outcomes in the following decades. Similarly, Fishback et al. (2020) conducted a detailed boundary analysis, examining socio-economic characteristics near C–D grade borders. They observed a decline in home values and an increase in black population shares on the D-side compared to the C-side.

The growing interest in environmental inequality has led to association-based studies concerning the redlining policy. Lane et al. (2022) revealed a consistent and nearly monotonic relationship between air pollutants and redlining grades, noting particularly an increase (over 50%) in NO$_2$ concentrations from A-graded to D-graded regions. The study also found that within each grade, disparities in air pollution exposure based on race and ethnicity continue to exist. This underscores the racially discriminatory impact of redlining on communities. Additionally, Jung et al. (2022) discovered that between 1998 and 2012, in New York City, schools in historically redlined regions saw smaller reductions in combustion-related air pollutants compared to others. However, the direct causal link between redlining policies and air pollution exposure remains uncertain, despite the apparent association.

In this paper, we revisit the data and apply methods from spatial causal inference to determine if the link between redlining and air pollution persists after accounting for spatial dependence and confounding variables. We link this historical redlining data with current air pollution concentrations, and assesses the potential long-term environmental effects of redlining policies (1935–1974) on present-day air pollution concentrations. Although the implementation and enforcement of redlining policies may have evolved over time, the HOLC maps that defined redlined neighborhoods remained fixed.

This analysis faces two key challenges. First, both redlining grades and air pollution concentrations exhibit spatial patterns, which must be carefully considered. Second, there is a risk of unmeasured confounding factors, particularly socio-economic status, that could influence both the historical redlining grades and current air pollution concentrations. In the following section, we outline our approach to addressing these challenges.

### 1.2 Spatial causal inference and unmeasured confounding

Addressing unmeasured confounding has become a major topic in causal inference. A unmeasured confounder could introduce bias into the estimated effect and lead to incorrect conclusions about the true causal relationship. There are many methods to adjust for unmeasured confounding, such as instrumental variables (Bound et al., 1995), negative controls (Lipsitch et al., 2010), latent and proxy variables (Kuroki and Pearl, 2014). These methods are not specifically designed for spatial data, but have been adopted to account for spatial unmeasured confounding in application studies, such as Davis et al. (2021), Shao et al. (2022), Giffin et al. (2021), Haschka et al. (2020), Tustin et al. (2017), and Jerzak et al. (2023).

Moreover, causal methods applied to complex spatial data have been drawing attention. A spatial confounder is a unmeasured confounder that contains spatial structure. The “blessing” of spatial confounder, compared with unstructured confounder, is that the spatial information may be used to capture some of the variability in the confounder, thus mitigating the bias (Gilbert et al., 2024). Dupont et al. (2022) developed method named “spatial+,” for cases when the treatment is spatially dependent but not fully determined by spatial location. A partial linear regression was used to adjust for spatial confounding. Guan et al. (2023) assumed a global-scale confounding (global relative to the treatment variable) and adjusted for confounding in the spatial domain by adding a spatially smoothed version of the treatment to the mean of the response variable. In spatial+ and the spectral adjustment, the treatment was assumed continuous. Other methods include region adjustment via spatial smoothing (Schnell and Papadogeorgou, 2020), distance adjusted propensity score matching (Papadogeorgou et al., 2019), spatial propensity-score (Davis et al., 2019), which have been reviewed in Reich et al. (2021).

There are challenges in the redlining data that cannot be addressed by existing methods. Social-economic status is arguably the most important confounding variable. Though some relevant data can be found in the US Census, it is dangerous to assume we can use them to fully account for social-economic status, thus potentially biasing causal interpretations. Moreover, the time lapse of 75 years between the policy action and the pollution measurement further obscures causal links. In addition, the existence of spatial correlation in all of treatment, outcome, proxies, and potentially the latent confounder, complicates the problem.

These challenges call for a method that addresses both latent confounding through proxies and unmeasured spatial confounding. Existing approaches, however, do not jointly leverage spatial structure and proxy variables to account for unobserved spatial confounders, even though proxy-based methods are well established in non-spatial causal analysis (Kong et al. (2022), Yang et al. (2024), and Miao et al. (2024)). This paper seeks to fill that gap.

### 1.3 Contributions and structure of the paper

Our study develops a novel latent framework for causal inference that accounts for both spatial and non-spatial confounding. We establish the identification of causal effects under broad assumptions, and use Bayesian MCMC to quantify uncertainty. Our method promises to enhance the validity of causal claims by rigorously adjusting for confounders. In the case study, we assesses the potential long-term environmental effects of redlining policies on present-day air pollution concentrations. The paper is organized as follows: Section 2 introduces the motivating data; Sections 3 and 4 present the statistical methods and their theoretical properties; Section 5 discusses computational aspects; Section 6 reports simulations; Section 7 applies the method to the motivating data; and Section 8 concludes.

## 2 DATA DESCRIPTION

The data for our study is drawn from multiple sources. We obtain the treatment variable, that is the redlining grades, from the Mapping Inequality Project (Nelson et al., 2023), including “A” for Desirable, “B” for Still Desirable, “C” for Declining, and “D” for Redlined.

For the outcome variables, we use annual average values of fine particulate matter (PM$_{2.5}$) and nitrogen dioxide (NO$_2$) concentrations in 2010. This year is selected because comprehensive air pollution monitoring for PM$_{2.5}$ and NO$_2$ began in the late 1990s, with sufficient data becoming available from 2010 onward (US EPA, 2024). The pollution data is derived from empirical models provided by the Center for Air, Climate, and Energy Solutions (CACES) (Kim et al., 2020). To address potential confounding, we incorporate variables from the 1940 US Census: unemployment rate, mean house rent, and percentage of Black population.

The geographical boundaries of the HOLC maps, the 1940 Census tracts, and the 2010 air pollution monitoring data differ. We merge these datasets by spatially overlapping them within the HOLC regions. Detailed methodologies for this spatial integration are available in Web Appendix A. After data cleaning, our final dataset includes 4079 regions across 69 cities and 27 states in the USA, covering about 20% of the 1940 US population.

Web Table S1 presents key summary statistics from the 1940 Census and 2010 air pollution data comparing redlined and non-redlined groups across all cities. Example maps for Atlanta, GA are shown in Web Figure S1. There is clearly spatial dependence in the pollution, redlining, and census variables. We observe higher mean values of NO$_2$, PM$_{2.5}$, unemployment rate, and percentage of Black population in areas with worse redlining grades. The percentage of Black population is zero-inflated, with approximately 5% of the observed values being zero. Conversely, mean house rent follows an opposite trend, with higher rents observed in non-redlined areas.

There is a substantial time gap between the redlining era (1935–1974) and the 2010 air pollution data. This introduces challenges such as potential attenuation of the redlining effects on air quality over time. Additionally, it complicates the identification and acquisition of confounders, particularly socio-economic status, which is a critical but debated concept among social scientists. To address this, we use data from the 1940 Census, including unemployment rates, housing conditions, and racial composition, as proxies for the underlying socio-economic status construct.

## 3 STATISTICAL METHODS

The data are drawn from *M* cities. City *i* is partitioned into $N_i$ regions. For region $j \in \lbrace 1, \ldots , N_i\rbrace$ in city *i*, the observed outcome, binary treatment, and *p* proxy variables are denoted by $Y_{ij} \in \mathcal {R}$, $A_{ij} \in \lbrace 0,1\rbrace$, and ${\bf W}_{ij} = (W_{ij1}, \ldots , W_{ijp})^\top \in \mathcal {R}^p$. While we present the model with a binary treatment, it can be readily extended to accommodate multi-level treatments (see Section 7). We posit two latent processes to capture confounding. The first is a non-spatial latent confounder process ${\bf U}_{ij} = (U_{ij1}, \ldots , U_{ijq})^\top \in \mathcal {R}^q$, which accounts for unobserved factors influencing both treatment and outcome. The second is a spatial process $Z_{ij} \in \mathcal {R}$, which explains dependence for nearby regions. Consider the model


(1) \begin{eqnarray*}
Y_{ij} &=& \alpha _y + \theta A_{ij} + \boldsymbol{\alpha }_{yu}^\top {\bf U}_{ij} + \alpha _{yz} Z_{ij} + \epsilon _{y,ij} ,
\end{eqnarray*}

(2) \begin{eqnarray*}
A_{ij} &=& \mathrm{I}(\alpha _a + \boldsymbol{\alpha }_{au}^\top {\bf U}_{ij} + \alpha _{az} Z_{ij} + \epsilon _{a,ij}>0),
\end{eqnarray*}

(3) \begin{eqnarray*}
{\bf W}_{ij} &=& \boldsymbol{\alpha }_w + \boldsymbol{\alpha }_{wu} {\bf U}_{ij} + \boldsymbol{\epsilon }_{w,ij},
\end{eqnarray*}

where the error terms $\epsilon _{y,ij}$, $\epsilon _{a,ij}$, and $\boldsymbol{\epsilon }_{w,ij}$ are independent and identically distributed with mean zero and variances $\sigma _y^2$, $\sigma _a^2$, and $\boldsymbol{\Sigma }_w = \mathrm{diag}(\sigma _{w_1}^2, \ldots , \sigma _{w_p}^2)$, respectively. Vectors $\boldsymbol{\alpha }_{au}$ and $\boldsymbol{\alpha }_{yu}$, both of length *q*, represent the coefficients for the relationships between the latent confounder ${\bf U}_{ij}$ and the treatment $A_{ij}$, and between ${\bf U}_{ij}$ and the outcome $Y_{ij}$, respectively. The matrix $\boldsymbol{\alpha }_{wu}$ is of dimension $p \times q$, representing the coefficients for the relationships between the proxy variables ${\bf W}_{ij}$ and the latent confounders ${\bf U}_{ij}$. Scalars $\alpha _{az}$ and $\alpha _{yz}$ represent the coefficients between the spatial process $Z_{ij}$ and the treatment $A_{ij}$, and between $Z_{ij}$ and the outcome $Y_{ij}$, respectively. The scalar $\theta$ is the treatment effect we aim to identify and estimate. Intercept terms include the vector $\boldsymbol{\alpha }_{w}$ and scalars $\alpha _{a}$ and $\alpha _{y}$.

With this design, we acknowledge the presence of latent confounders ${\bf U}_{ij}$ that can be captured through proxy variables ${\bf W}_{ij}$, and account for potential spatial confounding through the shared spatial process $Z_{ij}$. ${\bf U}_{ij}$ are assumed to follow ${\bf U}_{ij} \sim \mathcal {D}u(\boldsymbol{\mu }_u, \boldsymbol{\Sigma }_u)$, where $\boldsymbol{\Sigma }_u = \mathrm{diag}(\sigma _{u_1}^2, \ldots , \sigma _{u_q}^2)$ and $\mathcal {D}u(\cdot )$ denotes an arbitrary distribution with mean and finite variance; without loss of generality, the mean $\boldsymbol{\mu }_u$ can be set zero. The spatial latent variables are modeled using splines


(4) \begin{eqnarray*}
Z_{ij} = \sum _{l=1}^{L_{i}} \lambda _{il} B_{ijl}, \quad \lambda _{il} \sim \mathcal {D}_\lambda (0, \sigma _z^2),
\end{eqnarray*}

where $\mathcal {D}_\lambda (\cdot )$ means arbitrary distribution with mean and finite variance; $B_{ijl}$ denotes the *l*-th spline basis function integrated over regions *j* in city *i* (see Section 5), and $\lambda _{il}$ are the corresponding coefficients.

## 4 THEORETICAL PROPERTIES

We follow the potential outcomes framework (Rubin, 1976) and denote binary treatment as *A* and outcome as *Y*, then the potential outcomes given a treatment is $Y(a)$. We are interested in the average treatment effect ATE $=\mathbb {E}(\sum _{i=1}^{M}\sum _{j=1}^{N_i}\big \lbrace Y_{ij}(1)-Y_{ij}(0)\big \rbrace / (M \sum _{i=1}^{M} N_i))$. With appropriate assumptions, we show that $\theta$ in Equation (1) is the ATE and we can directly use $\widehat{\theta }$ as an ATE estimator.

Assumption 1 (SUTVA; Stable Unit Treatment Value Assumption): (1) the potential outcomes for any unit does not vary with the treatment assigned to other units; (2) there are no different versions of each treatment level leading to different potential outcomes.

Although our theoretical framework is presented for a binary treatment, it extends naturally to the multi-level case with HOLC grades A to D as distinct treatment levels. Here, the SUTVA requirement means that neighborhoods with the same grade are comparable, without substantively different versions of that treatment.

Assumption 2 (Latent Ignorability):
$A_{ij} \perp\perp Y_{ij}(a)| {\bf U}_{ij}, Z_{ij}$ for any *a*. In other words, ${\bf U}_{ij}$ and $Z_{ij}$ account for all confounders influencing treatment and outcome.

Assumption 3 (Latent Positivity):
$P(A_{ij}=a| {\bf U}_{ij}, Z_{ij}) > 0$ for any $a \in \lbrace 0, 1\rbrace$. That is, every unit has a non-zero probability of being assigned any treatment value.

Assumption 4 (Structural Causal Model): The data-generating process is as specified in Equations (1)–(4).

Note that the spline representation $Z_{ij}$ is an approximation of an underlying smooth spatial process $Z_{ij}^{*}$, not a literal assumption about the true data-generating mechanism. Under mild smoothness conditions, spline expansions provide a consistent and flexible approximation. The indicator function $I(\cdot )$ in Equation (2) can be relaxed. In addition, we assume the two latent process are independent $Z_{ij}\perp\perp {\bf U}_{ij}$.

Assumption 5 (Sufficient Condition for Factor Model): Let $\boldsymbol{\Lambda }= \boldsymbol{\alpha }_{wu}\boldsymbol{\Sigma }_{u|a}^{1/2}$, where $\boldsymbol{\Sigma }_{u|a}$ represents the conditional variance of ${\bf U}_{ij}$ given treatment $A_{ij} = a$. If any row of $\boldsymbol{\Lambda }$ is deleted, there remain two disjoint submatrices of rank *q*.

Assumption 5 ensures identification up to rotations (Anderson and Rubin, 1956), commonly known as the row-deletion property. A recent causal paper, Kang et al. (2025), adopts the same row-deletion framing and further explains that the condition implies each confounder should be correlated with at least three outcomes or treatments as an intuitive rule of thumb. This assumption is structural, and not directly testable using observed data.

Theorem 1: Under Assumptions (1)–(5), or by replacing Assumptions (3) and (5) with their continuous counterparts (3′) and (5′), the causal effect $\theta$ is identifiable.

Our results also apply to continuous treatments, with modifications to Assumptions 3 and 5. These continuous counterparts are: ASSUMPTION 3′: There exists a subset $\mathcal {A} \subseteq \mathbb {R}$ with positive Lebesgue measure such that the probability density $p(a \mid {\bf U}_{ij}, Z_{ij}) > 0$ for all $a \in \mathcal {A}$; ASSUMPTION 5′: *Let $\boldsymbol{\Lambda }= \boldsymbol{\alpha }_{wu}\boldsymbol{\Sigma }_u^{1/2}$. If any row of $\boldsymbol{\Lambda }$ is deleted, there remain two disjoint submatrices of rank *q**. Full derivations for both binary and continuous cases are provided in Web Appendix B.

## 5 COMPUTATIONAL DETAILS

### 5.1 Approximation of spatial confounders

We assume an independent spatial latent process for each city, and approximate spatial confounding $Z_{ij}$ by $Z_{ij} = \sum _{l=1}^{L_i} \lambda _{il} B_{ijl}$, where $B_{ijl}$ is the *l*-th pre-computed spline basis function for region *j* of city *i* and $\lambda _{il}$ is the corresponding coefficient. The number of basis function is taken to be $L_i = \lfloor r N_i \rfloor$ where *r* is the ratio of the number of basis functions to the number of regions in a city. The ratio *r* is selected by minimizing Watanabe-Akaike Information Criterion (WAIC) of the outcome model (Gelman et al., 2014).

To create spline basis functions for city *i*, we define the minimum bounding rectangle $\mathcal {G}_i$ that encompasses city *i*, and place a 100-by-100 grid of points within $\mathcal {G}_i$, denoted by coordinates $\boldsymbol{s}_{k}$ for $k = \lbrace 1,2,...,10000\rbrace$. Then we construct 2D cubic splines using this coordinates, denoted $b_{il}(\boldsymbol{s})$ for $l = \lbrace 1,...,L_i\rbrace$. Finally, within polygon $A_{ij}$, we integrate these splines over locations $\boldsymbol{s}_k \in A_{ij}$, resulting in the spline basis $B_{ijl} = \sum _k b_{il}(\boldsymbol{s}_{k}){\bf 1}(\boldsymbol{s}_k \in A_{ij}) / \sum _k {\bf 1}(\boldsymbol{s}_k \in A_{ij})$.

### 5.2 Bayesian Markov Chain Monte Carlo

We use Bayesian methods to incorporate uncertainties and address the inherent challenges in the complex data structure, including spatial and non-spatial latent variables, and zero-inflated proxies. We use a Markov chain Monte Carlo (MCMC) approach to sample from the joint posterior distribution of our model. Standard techniques for MCMC are employed, and uninformative priors are used when necessary. For simulation studies, we run single chain MCMC with 50 000 burn-in and 50 000 post burn-in iterations, with a thinning factor of 10. For real data analysis, we run single chain MCMC with 150 000 burn-in and 150 000 post burn-in, with a thinning factor of 10. Convergence is monitored using trace plots. Further details are provided in Web Appendix C.

## 6 SIMULATION STUDY

The objectives of the simulation study are to evaluate the performance of our model in terms of estimation and inference. We conduct the simulation using two settings: (1) creating data with simple grid geometry and predetermined parameters, and (2) creating data that closely resemble the redlining data. For each parameter setting, we randomly generate 100 datasets.

### 6.1 Data generation

We simulate data on 490 regions across 10 cities, arranged in simple 7-by-7 grids. Outcomes, treatments, and proxy variables are generated according to the structural model in Section 3. Six scenarios are considered: (1) a base case, (2) stronger proxy, (3) noisier outcome, (4) rougher spatial confounding, (5) weaker proxy, and (6) stronger latent confounding. Full details of the parameter values and equations are provided in Web Appendix D.

To generate data that closely resemble the redlining data, we use the geometry of the redlining data. For each dataset, we take a random subset cities from the real data such that there are at least 500 regions in a dataset. We define true parameters as the posterior parameter estimates from real Redlining data analysis (using $r=60\%$). There are two cases: (a) outcome of NO$_2$ and (b) outcome of PM$_{2.5}$.

### 6.2 Competing methods and metrics

We compare our method with two alternatives: (1) No adjustment for latent SES, removing latent process ${\bf U}_{ij}$ and proxy ${\bf W}_{ij}$ from the model; (2) Outcome Regression with Proxy, removing ${\bf U}_{ij}$ and moving proxy ${\bf W}_{ij}$ into the outcome regression as covariates. Both methods contains the spatial latent process $Z_{ij}$. We run models using spline ratios $r=\lbrace 0\%, 20\%, 40\%, 60\%, 80\%\rbrace$ to explore a broad range of complexities in the spatial latent processes, and select the best model based on WAIC.

We denote the true effect as $\theta ^{*}$. For each method, we denote the effect estimates as $\widehat{\theta }_t$ for simulation data set *t*, $t \in \lbrace 1,...,100\rbrace$, and $(L_t, U_t)$ as the corresponding credible intervals. We compute the following statistics: absolute bias $= 100^{-1}|\sum _{t=1}^{100}(\widehat{\theta }_t - \theta ^{*})|$, mean squared error (MSE) $= 100^{-1}\sum _{t=1}^{100}(\widehat{\theta }_t - \theta ^{*})^2$, and coverage probability $=100^{-1} \sum _{t=1}^{100} \mathbb {I} (\theta ^{*} \in [L_t, U_t])$.

### 6.3 Results

The simulation results are shown in Table 1, including the absolute bias (A.B.), mean squared error (MSE), coverage probability (%, C.P.), WAIC optimized over *r*, and the spline ratio (S.R.), each averaged over 100 simulations. Cases (1)–(6) use fully synthetic data, and cases (a) and (b) closely mimics the redlining data.

**TABLE 1:** Simulation results by cases: (1) base case, (2) stronger proxy, (3) noisier outcome, (4) rougher spatial confounding, (5) weaker proxy, (6) stronger confounder, (a) use posterior parameters and outcome is NO$_2$, (b) use posterior parameters and outcome is PM$_{2.5}$. In case (1), (2), (3), the true spline ratio is 40%; in case (4), (a), (b), the true spline ratio is 60%. The columns display the average absolute bias (A.B.) with standard deviation, mean square error (MSE) with standard deviation, coverage probability (C.P.), Watanabe-Akaike Information Criterion (WAIC), and the selected spline ratio (S.R.).

Case	Method	A.B.	MSE	C.P.	WAIC	S.R.
(1)	Latent Adjustment	0.003 (0.106)	0.011 (0.015)	95	1083	54
	Outcome Regr with Proxy	0.245 (0.088)	0.068 (0.043)	18	1115	44
	No Adjustment	1.197 (0.106)	1.445 (0.257)	0	1460	42
(2)	Latent Adjustment	0.003 (0.086)	0.007 (0.010)	94	1030	49
	Outcome Regr with Proxy	0.135 (0.078)	0.024 (0.022)	55	1038	44
	No Adjustment	1.198 (0.102)	1.446 (0.246)	0	1460	42
(3)	Latent Adjustment	0.010 (0.159)	0.025 (0.040)	95	1590	43
	Outcome Regr with Proxy	0.228 (0.125)	0.067 (0.060)	54	1580	37
	No Adjustment	1.170 (0.143)	1.388 (0.341)	0	1736	40
(4)	Latent Adjustment	0.012 (0.107)	0.011 (0.014)	95	110	66
	Outcome Regr with Proxy	0.243 (0.090)	0.067 (0.041)	19	1144	58
	No Adjustment	1.198 (0.103)	1.445 (0.249)	0	1485	60
(5)	Latent Adjustment	0.044 (0.166)	0.029 (0.043)	85	1087	59
	Outcome Regr with Proxy	0.412 (0.097)	0.179 (0.077)	2	1209	45
	No Adjustment	1.141 (0.080)	1.309 (0.177)	0	1482	35
(6)	Latent Adjustment	0.080 (0.178)	0.038 (0.058)	84	1093	60
	Outcome Regr with Proxy	0.485 (0.116)	0.248 (0.110)	1	1469	39
	No Adjustment	2.371 (0.196)	5.660 (0.934)	0	1992	35
(a)	Latent Adjustment	0.001 (0.154)	0.023 (0.036)	90	1874	69
	Outcome Regr with Proxy	0.051 (0.215)	0.052 (0.079)	78	1905	68
	No Adjustment	0.640 (0.124)	0.425 (0.158)	0	1967	69
(b)	Latent Adjustment	0.003 (0.040)	0.002 (0.002)	91	470	69
	Outcome Regr with Proxy	0.004 (0.063)	0.004 (0.005)	73	498	68
	No Adjustment	0.048 (0.032)	0.003 (0.003)	67	478	68

In cases (1)–(4), our method obtains a satisfying coverage probability and negligible absolute bias and MSE. By minimizing WAIC, on average our method selects a spline ratio *r* that is only slightly higher than the true ratio. Cases (5) and (6) showed a minor decline in performance, with increased positive bias and lower coverage probabilities. Our exploratory checks suggest that if the correct spline ratio of 60% had been used, the coverage probabilities for Cases (5) and (6) would have improved to 92% and 93%, respectively. That means, when the proxy ${\bf W}_{ij}$ is weak or the confounding effect of $U_{ij}$ is strong, challenges in model selection persist, leading to increased bias and reduced coverage probability.

In cases (a) and (b), the coverage probability is 90% for NO$_2$ and 91% for PM$_{2.5}$. Comparing across methods, our approach consistently produces coverage probabilities closest to the nominal level, along with lowest absolute bias and MSE. In sum, our method outperforms the alternatives across all evaluated metrics and simulation scenarios in Table 1.

For additional robustness checks, we provide further simulations in Web Table S2. These simulations explore model misspecification. In brief, our method performs poorly when the structural assumption 4 is violated. We provide full results in the Web Appendix.

## 7 REDLINING POLICY ANALYSIS

We apply our method to study the effect of redlining policy on air pollution exposure. To account for socio-economic status in the 1930s, we define three proxy variables: the Box-Cox transformed unemployment rate, the mean house rent, and the rank-based inverse normal transformed percent of Black population. We define the control group as grade A and the treatment groups as grades B, C, and D, denoting their respective treatment effects as “B-A,” “C-A,” and “D-A.” The outcomes are PM$_{2.5}$ and NO$_2$ concentrations in 2010, and we fit our model separately for each pollutant. All proxy variables and outcomes are centered by their mean per city before fitting the model. The model in Equation (1)–(3) is extended to have three binary treatment variable by adding a spatial term for each treatment variable. In addition to the constant causal effect model discussed in Section 3, we develop a random effect model, assuming that causal effects vary by city and follows i.i.d.. Further details on multiple treatments and random effects can be found in Web Appendix C.

### 7.1 Results

**FIGURE 1:**
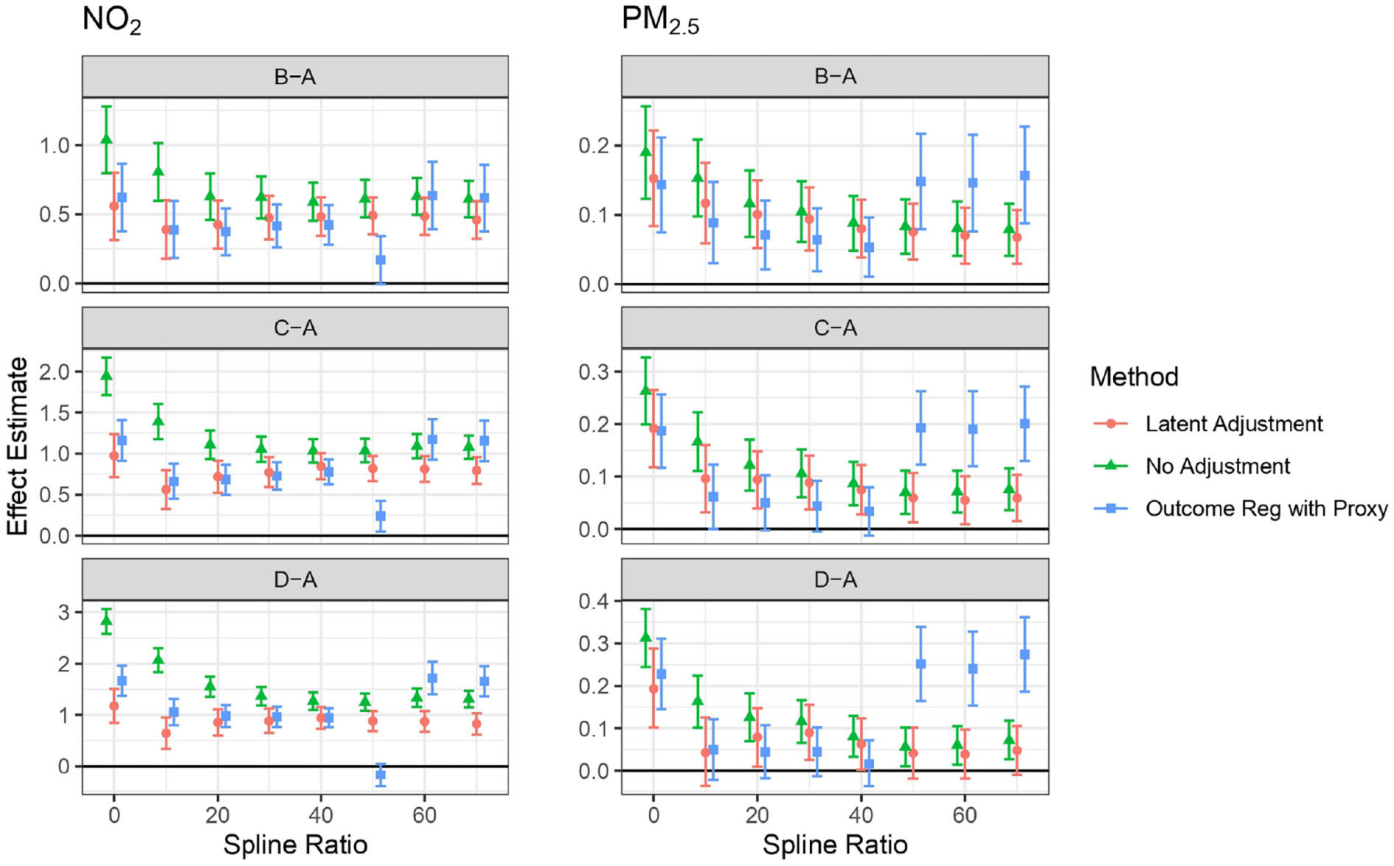
Redlining effect estimates and 95% credible intervals. The left panel shows results for NO$_2$ (ppb) and the right panel for PM$_{2.5}$ ($\mu$g/m$^3$). Estimates are shown for different redlining grades relative to grade A (“B-A,” “C-A,” “D-A”) under varying spline ratios.

Figure 1 shows the posterior distribution of the long-term effects of redlining policies on air pollution exposure. The estimates stabilize as the spline ratio increases for the Latent Adjustment and No Adjustment methods, indicating robustness across these approaches. However, the Outcome Regression with Proxy method shows greater variability, suggesting potential sensitivity to the spline choice.

For most treatment groups and pollutants, the estimated effects are significant and positive, indicating that redlining had a harmful impact on air quality under our modeling assumptions. Specifically, at $r=60\%$, the estimated effect for NO$_2$ is 0.48 ppb (95% CI: 0.35 to 0.62) for “B-A,” 0.81 (0.65 to 0.96) for “C-A,” and 0.87 (0.67 to 1.08) for “D-A.” The estimated effect for PM$_{2.5}$ is 0.07 $\mu g/m^3$ (95% CI: 0.03 to 0.11) for “B-A,” 0.06 (0.01 to 0.10) for “C-A,” and 0.04 (-0.02 to 0.10) for “D-A.” This differs from the raw (associational) difference between all treatment groups (B, C, D) and control group A, which is 2.48 ppb for NO$_2$ and 0.26 $\mu$g/m$^3$ for PM$_{2.5}$. Overall, the Latent Adjustment method provides stable estimates, while the Outcome Regression with Proxy method exhibits substantial variability. If our model correctly represents the data-generating process, the No-Adjustment method tends to overestimate the effect for NO$_2$.

To place these modest concentration differences in context, we translated them into cumulative excess mortality (Web Appendix E, Web Table S5). For example, in “D versus A” comparisons, the estimated increase of 0.87 ppb NO$_2$ and 0.04 $\mu$g/m$^3$ increase in PM$_{2.5}$ corresponds to approximately 15 915 excess deaths (95% CI: 5642 to 28 039) over 40 years across the exposed population. These calculations highlight that even small pollutant increases, when sustained over decades and scaled to populations, may translate into meaningful public-health burdens.

Web Figure S4 demonstrates that Latent Adjustment achieves lowest WAIC values compared to the No Adjustment and Outcome Regression with Proxy. There is a rapid decrease in WAIC values until spline ratio *r* reaches approximately 60%. Given the stable estimates observed in Figure 1, we will highlight results at $r=60\%$ for the remainder of this paper.

We apply a random effect model with $r=60\%$, where the results of the constant effects model had stabilized over a wide range of *r* values. For NO$_2$, the estimated average treatment effects across cities are 0.14 ppb (95% CI: -0.04 to 0.31) for “B-A,” 0.37 (0.16 to 0.57) for “C-A,” and 0.50 (0.22 to 0.79) for “D-A.” These estimates suggest an increasing impact of redlining on NO$_2$ exposure across treatment levels, with stronger effects observed for more heavily redlined areas. For PM$_{2.5}$, the estimated average treatment effects cross cities are 0.04 $\mu g/m^3$ (95% CI: -0.02 to 0.09) for “B-A,” 0.03 (-0.03 to 0.09) for “C-A,” and -0.01 (-0.08 to 0.07) for “D-A.” These results indicate no strong evidence of a redlining effect on PM$_{2.5}$, with credible intervals spanning zero for all treatment levels. Overall, the random effects model confirms a statistically significant and increasing effect of redlining on NO$_2$ exposure, while the effects on PM$_{2.5}$ remain weak and inconclusive.

**FIGURE 2:**
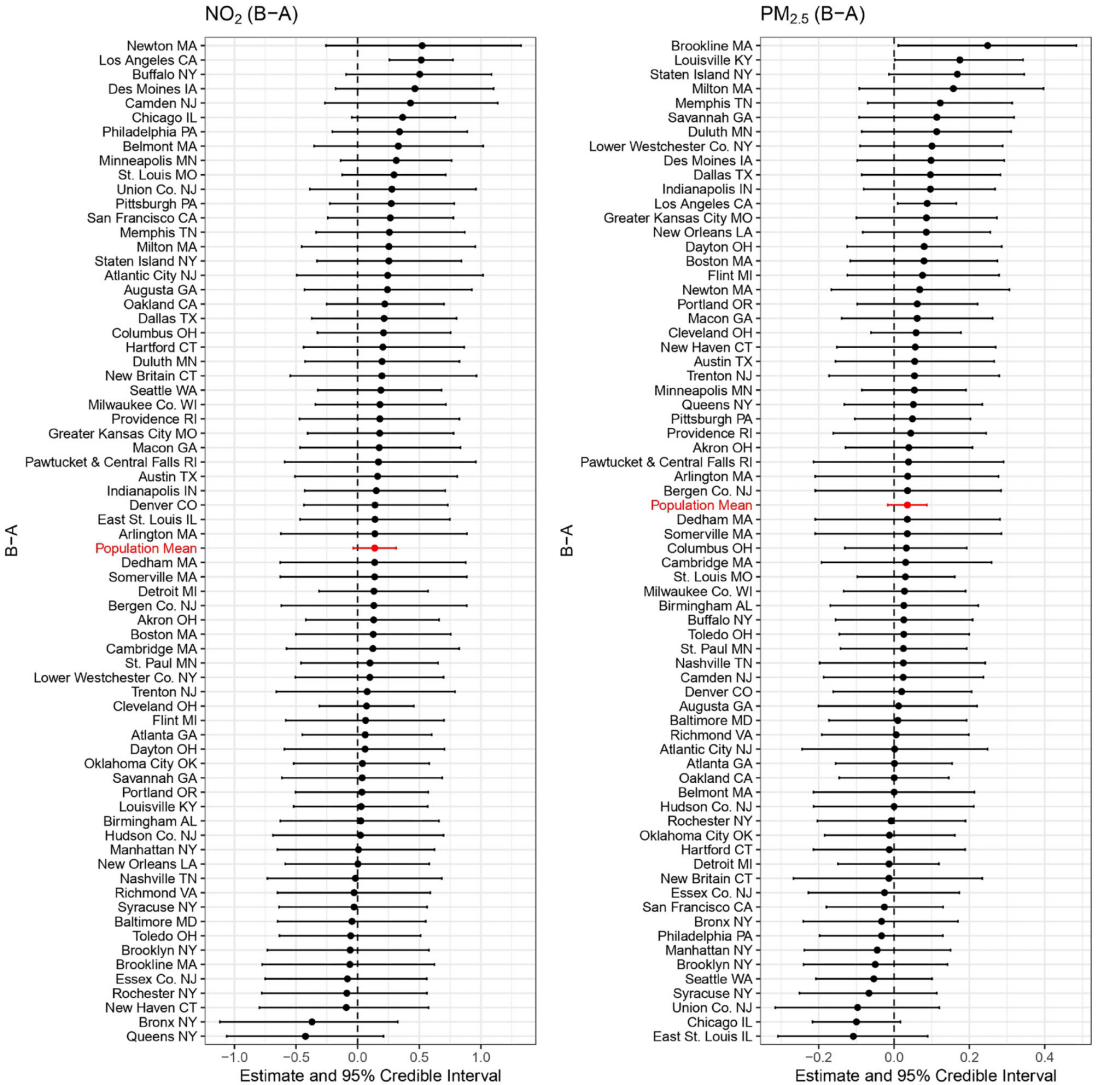
Posterior estimates of the long-term effects of “B-A” on NO$_2$ (left) and PM$_{2.5}$ (right) concentrations across 69 cities. Each city is represented by its posterior mean and 95% credible interval.

**FIGURE 3:**
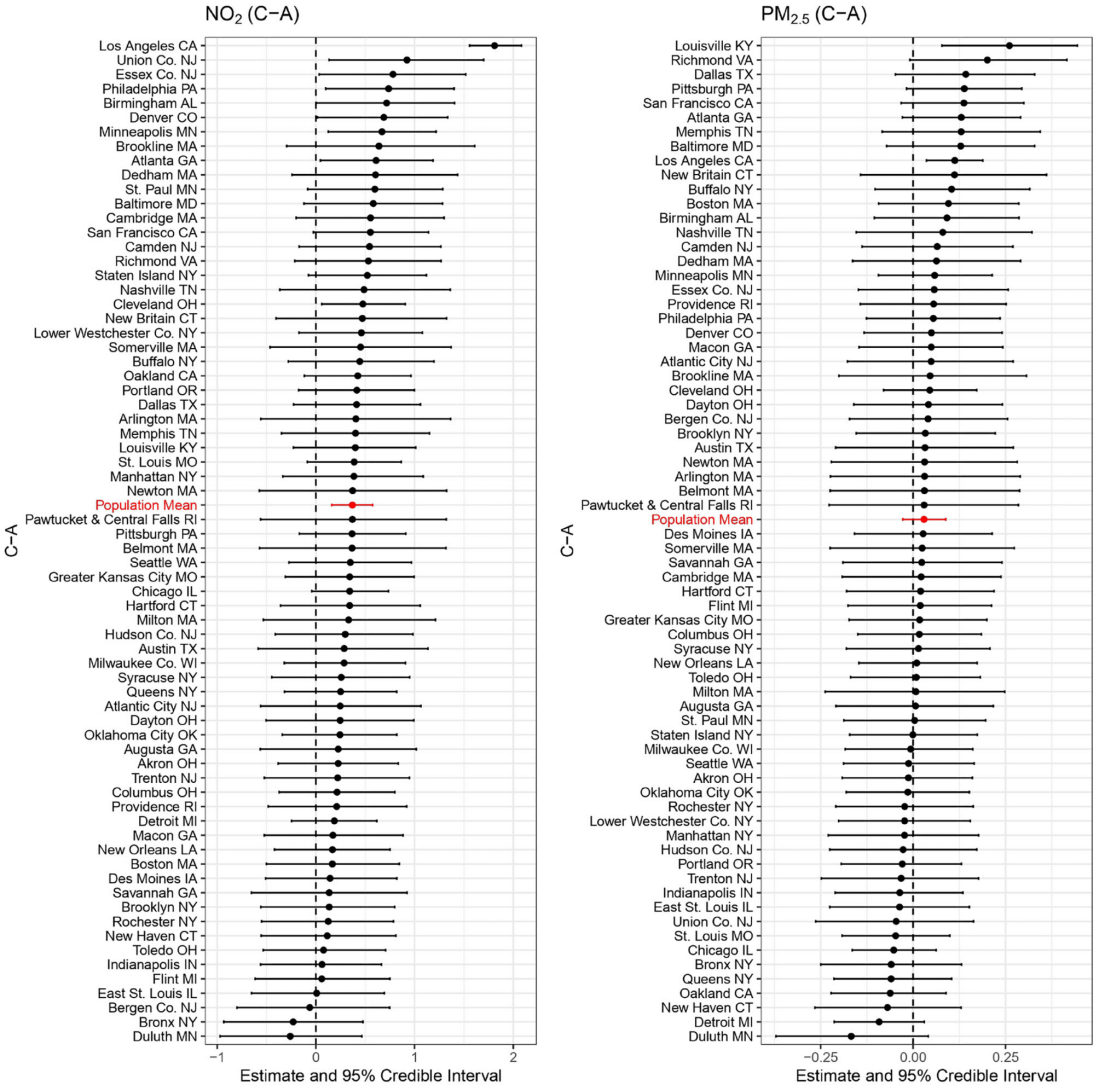
Posterior estimates of the long-term effects of “C-A” on NO$_2$ (left) and PM$_{2.5}$ (right) concentrations across 69 cities. Each city is represented by its posterior mean and 95% credible interval.

**FIGURE 4:**
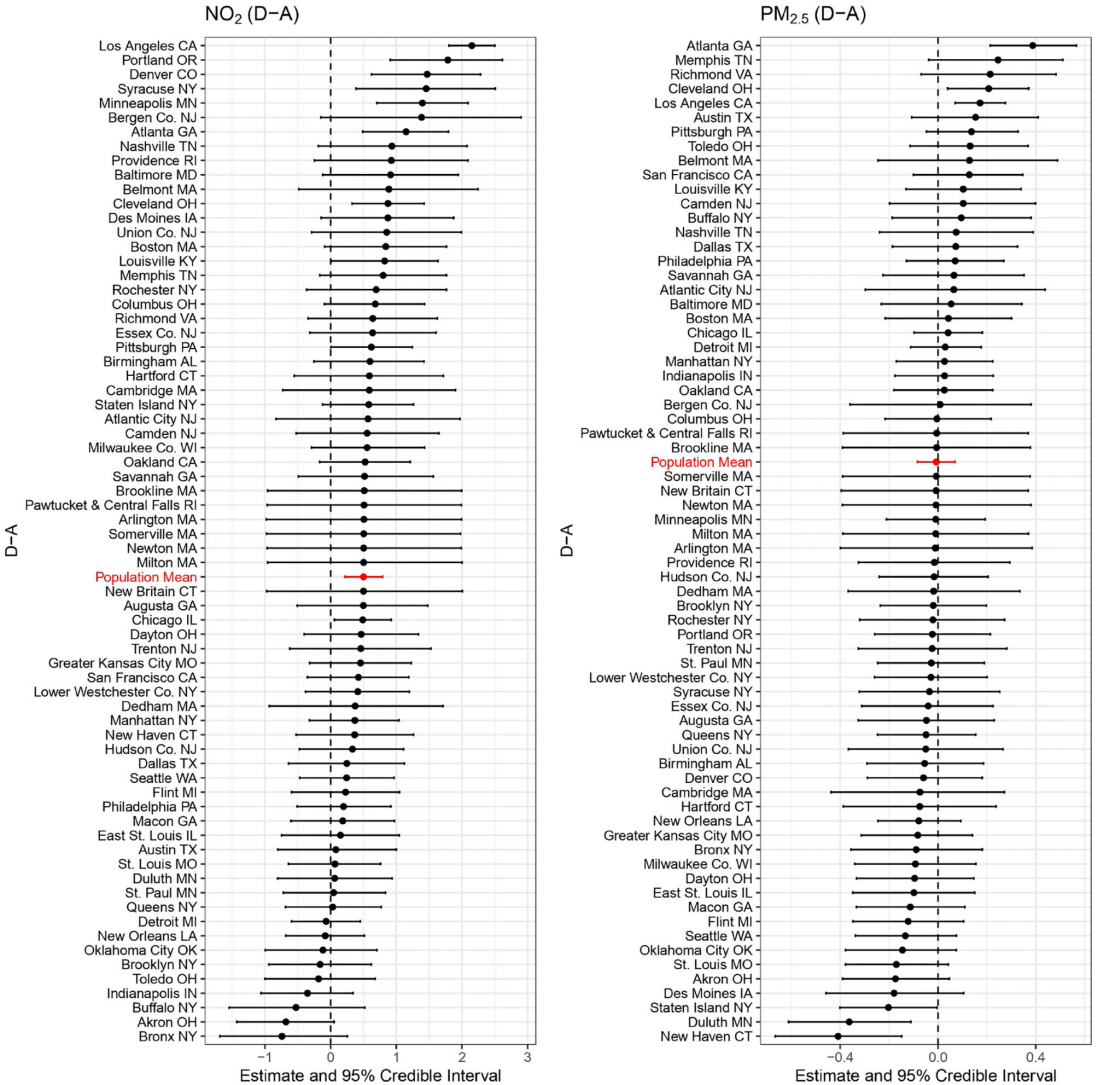
Posterior estimates of the long-term effects of “D-A” on NO$_2$ (left) and PM$_{2.5}$ (right) concentrations across 69 cities. Each city is represented by its posterior mean and 95% credible interval.

As shown in Figures 2, 3, and 4, several cities exhibit strong evidence of a harmful effect on NO$_2$ among different treatment groups (“B-A,” “C-A,” “D-A”), including Los Angeles, CA; Philadelphia, PA; Minneapolis, MN; Denver, CO; Atlanta, GA; Portland, OR; and Cleveland, OH. No cities show evidence of a protective effect for NO$_2$. For PM$_{2.5}$, the effects are generally weaker, but some cities still show significant disparities. Los Angeles, CA, and Atlanta, GA exhibit strong evidence of a harmful effect at the “D-A” comparison, while New Haven, CT, and Duluth, MN, show strong evidence of a protective effect. Notably, Los Angeles and Atlanta consistently show the strongest harmful effects for both NO$_2$ and PM$_{2.5}$, highlighting the persistent environmental impact of redlining in these cities.

**FIGURE 5:**
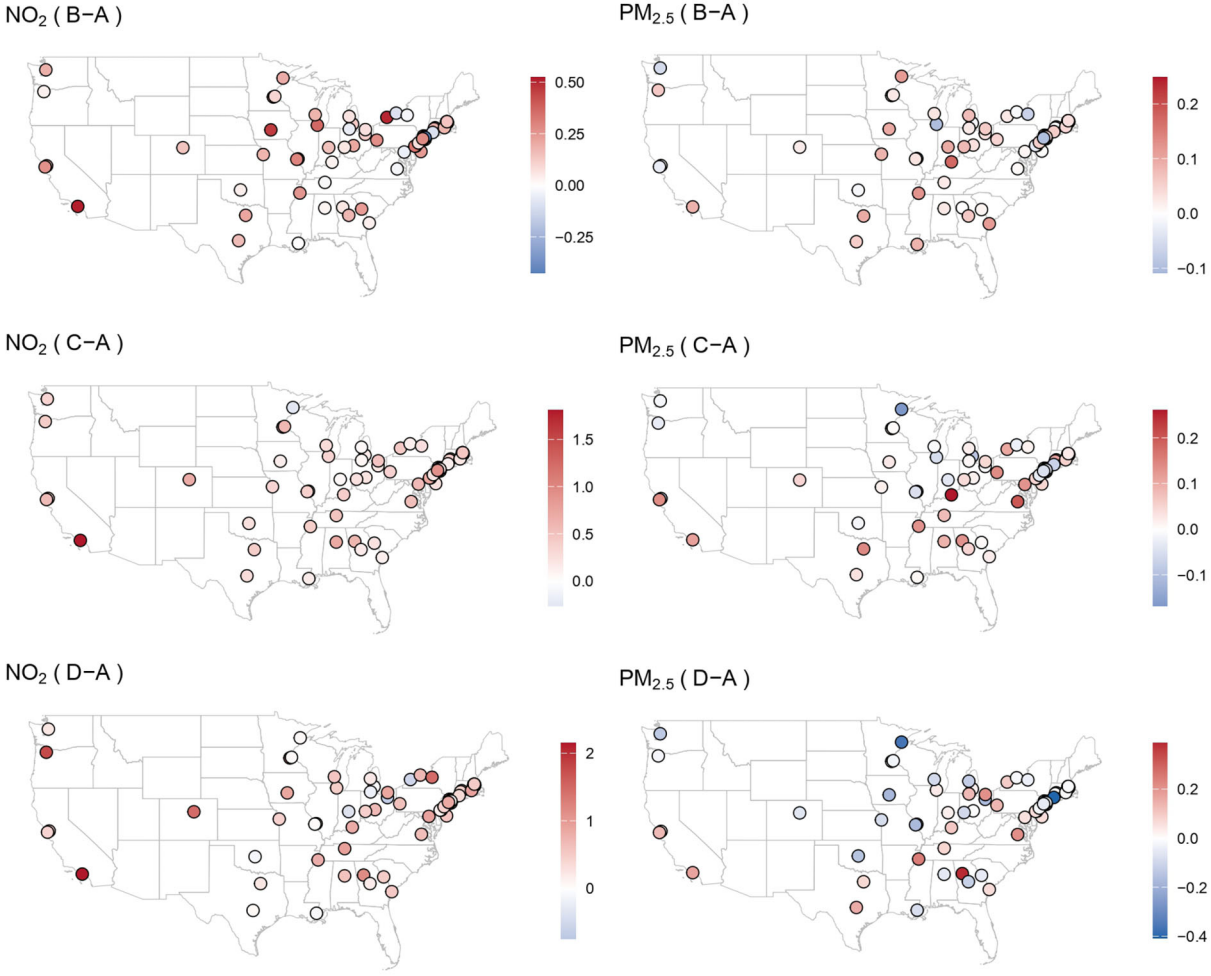
Mapping posterior estimates of the random effects across 69 cities. Harmful effects are represented by red and protective effects by blue, regardless of significance.

The spatial distribution of the long-term effects across 69 cities, as depicted in Figure 5, reveals distinct geographic patterns. For PM$_{2.5}$, the harmful effects, indicated by red dots, are predominantly concentrated along the East Coast and in certain Midwestern and Western cities. Conversely, protective effects, represented by blue dots, are more apparent in central and northern cities. The relationship between these spatial patterns and urban development and population trends warrants further investigation. For NO$_2$, the spatial distribution of harmful effects is much broader, encompassing a wide range of geographic regions.

In Web Figure S3 and Web Table S3, we confirm that the overlap and positivity assumptions are met, ensuring a solid foundation for causal inference. Web Table S4 demonstrates that we identify the latent $U_{ij}$ representing socio-economic status (SES) in the expected manner. A higher value in latent factor $U_{ij}$ indicates lower socio-economic status: $U_{ij}$ is associated with a higher unemployment rate, lower house rent, higher percentage of Black population, higher probability of being redlined, and higher air pollution concentrations.

## 8 DISCUSSION

We estimate the long-term causal effects of redlining policies (1935-1974) on present-day NO$_2$ and PM$_{2.5}$ air pollution concentrations in 69 cities across 27 US states. We found strong evidence suggesting harmful causal effects of redlining policies on NO$_2$ concentrations, with an estimated effect of 0.48 ppb (95% CI: 0.35 to 0.62) for “B-A,” 0.81 (0.65 to 0.96) for “C-A,” and 0.87 (0.67 to 1.08) for “D-A,” even 36 years after the policy ended. We find evidence of weak harmful effects on PM$_{2.5}$ concentrations after adjusting for unmeasured confounding, with an estimated effect of 0.07 $\mu$g/m$^3$ (95% CI: 0.03 to 0.11) for “B-A,” 0.06 (0.01 to 0.10) for “C-A,” and 0.04 (-0.02 to 0.10) for “D-A.” These findings suggest that redlining has had a more pronounced impact on NO$_2$ concentrations. NO$_2$ and PM$_{2.5}$ pollutants originate from different sources [US EPA (2023a), US EPA (2023b)]. A potential explanation for the disparity in impacts between NO$_2$ and PM$_{2.5}$ may lie in highway vehicles, which is the primary contributor to NO$_2$. Highway vehicles could act as a mediating factor between redlining policies and NO$_2$ exposure. Although the estimated concentration increases are modest and close to the scale of exposure model error, our mortality translation suggests they may nonetheless accumulate to non-trivial population health consequences when sustained over decades (Web Table S5).

To explore the variance of causal effects, we revise the model to include random effects. For NO$_2$, the population mean from the random effects model are 0.14 ppb (95% CI: -0.04 to 0.31) for “B-A,” 0.37 (0.16 to 0.57) for “C-A,” and 0.50 (0.22 to 0.79) for “D-A.” Most cities present harmful effects, although only a few are statistically significant. For PM$_{2.5}$, the population mean from the random effects model are 0.04 $\mu g/m^3$ (95% CI: -0.02 to 0.09) for “B-A,” 0.03 (-0.03 to 0.09) for “C-A,” and -0.01 (-0.08 to 0.07) for “D-A.” The harmful effects are predominantly concentrated along the East Coast and in certain Midwestern and Western cities. This pattern aligns well with the early urbanized areas of the 1930s and 1940s. Conversely, protective effects are more apparent in middle and northern cities, which largely urbanized during the Great Migration of the 1960s.

To our knowledge, this is the first study to investigate the causal effect of redlining policies on air pollution concentrations and one of the earliest to explore the causal effect of redlining policies on environmental risk exposure. The key strengths of this study are the following: (1) we conduct exhaustive adjustment for potential unmeasured confounding. We adjust for city-level confounders, spatial confounders using spatial splines, and confounder of socio-economic status using proxy variables. Our causal estimates are an order of magnitude smaller than recent association-based findings (Lane et al., 2022). (2) We prove the identification of causal effect given the data generating process modeled. (3) We quantify uncertainty using Bayesian MCMC. (4) We conduct intensive simulation study to demonstrate the performance on estimation and inference of our method over other currently available bias correction methods.

Our study has several limitations in data and modeling. First, our study covers only 69 cities. Historically, more cities were redlined (Nelson et al., 2023). The covered 69 cities might be the most urbanized, considering that they are covered in the 1940 US census while others are not. This suggests that our study may not be representative of the entire redlined population. Second, our analysis relies on assumptions for causal identification. The no-interference assumption may be restrictive, as HOLC grades in one neighborhood could influence nearby air quality through pollutant transport and broader socio-environmental mechanisms. We interpret our estimates as direct effects, while acknowledging that some spillovers may persist. Assessing such spillover effects more explicitly is an important direction for future work. Another limitation concerns Assumption 4, which posits a specific structural causal model. While this assumption is difficult to verify and violations can lead to poor performance (as shown in our simulations), the fact that our observed proxies for socio-economic status are linearly related to each other and that their associations with pollution outcomes do not appear to grossly violate linearity provides indirect support for its plausibility in the redlining context (Web Figures S6, S7). Nonetheless, we emphasize this as a limitation of our approach.

## Supplementary Material

ujaf173_Supplemental_Files Web Appendices (A–E), Web Tables (S1–S5), and Web Figures (S1–S7) referenced in Sections 2 and 4–8, as well as the code used to implement the proposed methods, are available with this paper at the Biometrics website on Oxford Academic.

## Data Availability

The data for redlining policy analysis are available with this paper in the Supplementary Materials.
