# Supplementary material for: Estimating the causal effect of redlining on present-day air pollution
Source: Biometrics. 2026 Jan 15;82(1):ujaf173. doi: 10.1093/biomtc/ujaf173 (PMC12805554; doi:10.1093/biomtc/ujaf173)
Supplement: ujaf173_Supplemental_Files — Web Appendices (A–E), Web Tables (S1–S5), and Web Figures (S1–S7) referenced in Sections 2 and 4–8, as well as the code used to implement the proposed methods, are available with this paper at the Biometrics website on Oxford Academic. [file ujaf173_supplemental_files.zip › Supplementary_Materials_for_Estimating_the_Causal_Effect_of_Redlining_on_Present-day_Air_Pollution.pdf]

Supplementary Materials for Estimating the Causal Effect of Redlining on Present-day Air Pollution by Xiaodan Zhou, Shu Yang, Brian J. Reich

Web Appendix A: Additional details of data cleaning

Our data cleaning process integrates multiple sources to create a coherent dataset for analysis.

Redlining Data: The mapping Inequality Project has digitized the 1937 'residential security maps' created by the Home Owners' Loan Corporation (HOLC) (https://dsl.richmond.edu/panorama/redlining/data). This digitized data is invaluable for the redlining policy evaluation, particularly given the challenges of data collection in the 1930s due to technological limitations. We obtained the redlining data version in September 2022. This dataset includes digitized HOLC maps for 202 cities. To align with the 2010 census boundaries, we reformat these maps to match the 2010 census geography.

1940 Census Data: The 1940 census data is sourced from the National Historical Geographic Information System (NHGIS) (https://data2.nhgis.org/main). This dataset is selected because it is the closest census following the implementation of the redlining policies and offers a broader coverage of redlined cities compared to the 1930 census. In the web page, we select GEOGRAPHIC LEVELS as TRACT, YEARS as 1940, and downloaded the data with geographic boundaries based on the 2008 TIGER/Line files.

Unemployment rate is calculated as the ratio of the unemployed population to the total labor force. For home rent, the census data provides 'gross monthly rent by homes' in categorical buckets (<\$5, \$5−\$6, ..., \$75−\$99, \$100+). We use the midpoint of each category to compute a count-weighted mean home rent. The percentage of Black Population is the ratio of self-identified Black individuals to the total population.

Air Pollution Data: The raw $PM_{2.5}$ and NO_2 data for 2010 at the census tract level were retrieved from the Center for Air, Climate, and Energy Solutions (CACES).

Spatial Join and Missing Values: We merge these datasets using a spatial join method. For population counts, we assume an even spatial distribution and applied area-based weighting. Similarly, for rent and air pollution data, we use area weights to join these layers spatially. After the spatial join, we identify four regions with missing values for mean home rent. These are imputed using the average of the available non-missing values at the city level. The merged data are accessible at https://github.com/xiaodan-zhou/Air_Pollution_Redlining.

Data Representation: The original HOLC dataset contains 8,878 regions across 202 cities; 5,264 of the regions in 69 cities are fully or partially overlapped by the 1940 Census. We focus on 4,079 regions in these 69 cities that overlapped with both the 1940 census data and the 2010 air pollution data. Specifically, of all HOLC regions, 80.3% have at least 98% of their area covered by the 1940 census; 86.1% have at least 90% of their area covered by the 1940 census. This cleaned dataset captures approximately 25.5 million individuals, representing 20% of the 1940 U.S. population, thus providing a robust basis for our analysis.

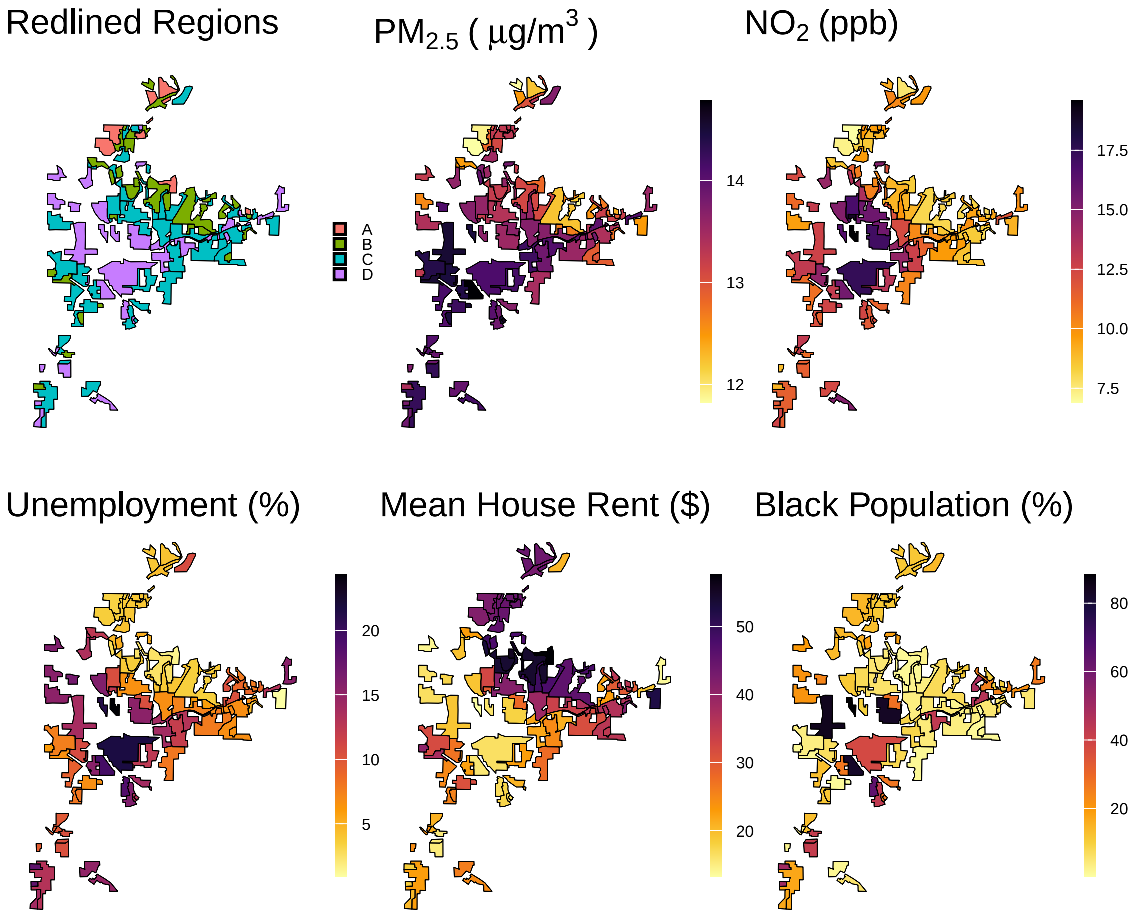

Web Figure S1: Data for Atlanta, GA. The first row shows the maps of redlined regions and non-redlined regions, and $PM_{2.5}$ and NO_2 levels. Second row shows unemployment rate, mean (monthly) house rent, and percent of Black population.

Web Table S1: Mean and standard error (in parentheses) by grade ranging from 'A' for 'Desirable' to 'D' for 'Redlined', pooled across cities.

	A	B	C	D
Count	437	1032	1618	992
2010 NO_2 (ppb)	11.13 (3.81)	12.84 (4.43)	14.57 (4.94)	15.20 (5.08)
2010 $PM_{2.5}$ ($\mu g/m^3$)	10.63 (2.11)	10.82 (2.07)	10.97 (1.94)	11.10 (1.93)
1940 Unemployment (%)	6.71 (3.90)	9.10 (4.27)	12.28 (4.97)	19.05 (7.55)
1940 Mean House Rent ($)	51.19 (14.17)	44.17 (11.87)	38.06 (10.24)	29.56 (9.07)
1940 Black Population (%)	2.45 (6.70)	1.93 (5.86)	3.09 (8.52)	12.98 (21.78)

Web Appendix B: Derivations

Identification Strategy: We discuss identifiability of model parameters in two scenarios, when the treatment is continuous and binary, respectively. When the treatment is binary, we obtain

$$\text{Cov}(\mathbf{W}_{ij}|A_{ij}) = \boldsymbol{\alpha}_{wu}\boldsymbol{\Sigma}_{u|a}\boldsymbol{\alpha}_{wu}^\top + \boldsymbol{\Sigma}_w, \quad (5)$$
$$\text{Cov}(\mathbf{W}_{ij}, Y_{ij}|A_{ij}) = \boldsymbol{\alpha}_{wu}\boldsymbol{\Sigma}_{u|a}\boldsymbol{\alpha}_{yu}, \quad (6)$$
$$E(\mathbf{W}_{ij}|A_{ij}) = \alpha_a + \boldsymbol{\alpha}_{wu}E(\mathbf{U}_{ij}|A_{ij}), \quad (7)$$
$$E(Y_{ij}|A_{ij}) = \theta A_{ij} + \boldsymbol{\alpha}_{yu}E(\mathbf{U}_{ij}|A_{ij}). \quad (8)$$

In Equation (5), let $\boldsymbol{\Lambda} := \boldsymbol{\alpha}_{wu}\boldsymbol{\Sigma}_{u|a}^{1/2}$ of shape $p \times q$. We add Assumption 5, which is a strong and sufficient condition and implies $p \geq 2q + 1$. With Assumption 5 and by applying Theorem 5.1 of Anderson and Rubin (1956), then $\boldsymbol{\Lambda}$ is identified up to rotations from the right under certain sufficient conditions. Specifically, $\boldsymbol{\alpha}_{wu}\boldsymbol{\Sigma}_{u|a}^{1/2}$ is identified up to multiplication on the right by an orthogonal matrix $\mathbf{R}_1$, so any admissible value for $\boldsymbol{\Lambda}$ can be written as $\boldsymbol{\Lambda}^* = \boldsymbol{\Lambda}\mathbf{R}_1$ with an arbitrary orthogonal matrix $\mathbf{R}_1$ of shape $q \times q$ (Miao et al. (2023), Kang et al. (2025)).

Plugging $\boldsymbol{\Lambda}$ into Equation (6), it becomes a linear system with p equations and q unknowns. $\boldsymbol{\Sigma}_{u|a}^{1/2}\boldsymbol{\alpha}_{yu}$ is identified up to multiplication on the left by $\mathbf{R}_1^\top$. Similarly, plugging $\boldsymbol{\Lambda}$ into Equation (7), then $E(\mathbf{U}_{ij}|A_{ij})$ is identified up to multiplication on the left by $\mathbf{R}_1^\top\boldsymbol{\Sigma}_{u|a}^{-1/2}$. Finally in Equation (8), $\boldsymbol{\alpha}_{yu}^\top E(\mathbf{U}_{ij}|A_{ij})$ has been identified since it can be expressed by $(\boldsymbol{\alpha}_{yu}^\top\boldsymbol{\Sigma}_{u|a}^{1/2}\mathbf{R}_1)(\mathbf{R}_1^\top\boldsymbol{\Sigma}_{u|a}^{-1/2}E(\mathbf{U}_{ij}|A_{ij}))$, which are two components that have been identified. Consequently, the causal effect θ in Equation (8) can be uniquely identified. When spatial confounding Z exists, we approximate it by B-splines (see details in Section 5.1). Equation (6) and (8) will be updated as below, while the method proof and conclusion remain the same.

$$\text{Cov}(\mathbf{W}_{ij}, Y_{ij}|A_{ij}) = \boldsymbol{\alpha}_{wu}\boldsymbol{\Sigma}_{u|a}\boldsymbol{\alpha}_{yu} + \text{Cov}(\mathbf{W}_{ij}, Z_{ij}|A_{ij})\alpha_{yz},$$
$$E(Y_{ij}|A_{ij}) = \theta A_{ij} + \boldsymbol{\alpha}_{yu}E(\mathbf{U}_{ij}|A_{ij}) + \alpha_{yz}E(Z_{ij}|A_{ij}).$$

When the treatment is continuous, we obtain

$$\text{Cov}(\mathbf{W}_{ij}) = \boldsymbol{\alpha}_{wu}\boldsymbol{\Sigma}_u\boldsymbol{\alpha}_{wu}^\top + \boldsymbol{\Sigma}_w, \quad (9)$$

$$\text{Cov}(\mathbf{W}_{ij}, Y_{ij}) = \text{Cov}(\mathbf{W}_{ij}, A_{ij})\theta + \boldsymbol{\alpha}_{wu}\boldsymbol{\Sigma}_u\boldsymbol{\alpha}_{yu}. \tag{10}$$

In the first equation, let $\boldsymbol{\Lambda} := \boldsymbol{\alpha}_{wu}\boldsymbol{\Sigma}_u^{1/2}$ of shape $p \times q$. We add Assumption 5', then by applying Lemma 5.1 and Theorem 5.1 of Anderson and Rubin (1956), $\boldsymbol{\Lambda}$ is identified up to multiplication on the right by orthogonal matrix $\mathbf{R}$. In the second equation, denote $\Xi = \boldsymbol{\Sigma}_u^{1/2}\boldsymbol{\alpha}_{yu}$, resulting in a linear system with p equations and $(1+q)$ unknowns of θ and Ξ. With the same Theorem 5.1 by Anderson and Rubin (1956), $p \geq 2q+1$ holds, therefore, these equations are over-determined and can be solved uniquely for θ using $\text{Cov}(\mathbf{W}_{ij}, Y_{ij}) = \text{Cov}(\mathbf{W}_{ij}, A_{ij})\theta + \boldsymbol{\Lambda}^*\Xi$.

Derivation of Equation (5) and (6) for binary treatment: Without loss of generality, we set intercepts $\boldsymbol{\alpha}_w = 0, \alpha_a = 0, \alpha_y = 0$, and set $\boldsymbol{\mu}_u = 0$ for latent factors. The models conditioning on the treatment can be formatted as below,

$$\left[\begin{array}{c|c} \mathbf{W}_{ij} & \\ Y_{ij} & A_{ij} \\ Z_{ij} & \end{array}\right] = \begin{bmatrix} \boldsymbol{\Gamma} & 0 & 0 \\ 0 & 0 & \alpha_{yz} \\ 0 & 0 & 0 \end{bmatrix} \left[\begin{array}{c|c} \mathbf{W}_{ij} & \\ Y_{ij} & A_{ij} \\ Z_{ij} & \end{array}\right] + \begin{bmatrix} \boldsymbol{\alpha}_{wu} \\ \boldsymbol{\alpha}_{yu}^\top \\ \boldsymbol{\alpha}_{zu|a}^\top \end{bmatrix} \left[\begin{array}{c|c} \mathbf{U}_{ij} & A_{ij} \end{array}\right] + \begin{bmatrix} \boldsymbol{\epsilon}_w \\ \epsilon_y \\ \epsilon_z \end{bmatrix}$$

Let $\mathbf{B} = \begin{bmatrix} \boldsymbol{\Gamma} & 0 & 0 \\ 0 & 0 & \alpha_{yz} \\ 0 & 0 & 0 \end{bmatrix}$, then $(\mathbf{I}-\mathbf{B})^{-1} = \begin{bmatrix} \mathbf{I}-\boldsymbol{\Gamma} & 0 & 0 \\ 0 & 1 & -\alpha_{yz} \\ 0 & 0 & 1 \end{bmatrix}^{-1} = \begin{bmatrix} (\mathbf{I}-\boldsymbol{\Gamma})^{-1} & 0 & 0 \\ 0 & 1 & \alpha_{yz} \\ 0 & 0 & 1 \end{bmatrix}$

$$\text{Cov}\left[\begin{array}{c|c} \mathbf{W}_{ij} & \\ Y_{ij} & A_{ij} \\ Z_{ij} & \end{array}\right]$$

$$= \begin{bmatrix} (\mathbf{I}-\boldsymbol{\Gamma})^{-1} & 0 & 0 \\ 0 & 1 & \alpha_{yz} \\ 0 & 0 & 1 \end{bmatrix} \begin{bmatrix} (\boldsymbol{\alpha}_{wu}\boldsymbol{\Sigma}_{u|a}\boldsymbol{\alpha}_{wu}^\top + \boldsymbol{\Sigma}_w) & \boldsymbol{\alpha}_{wu}\boldsymbol{\Sigma}_{u|a}\boldsymbol{\alpha}_{yu} & \boldsymbol{\alpha}_{wu}\boldsymbol{\Sigma}_{u|a}\boldsymbol{\alpha}_{zu|a} \\ \boldsymbol{\alpha}_{yu}^\top\boldsymbol{\Sigma}_{u|a}\boldsymbol{\alpha}_{wu}^\top & (\boldsymbol{\alpha}_{yu}^\top\boldsymbol{\Sigma}_{u|a}\boldsymbol{\alpha}_{yu} + \sigma_y^2) & \boldsymbol{\alpha}_{yu}^\top\boldsymbol{\Sigma}_{u|a}\boldsymbol{\alpha}_{zu|a} \\ \boldsymbol{\alpha}_{zu|a}^\top\boldsymbol{\Sigma}_{u|a}\boldsymbol{\alpha}_{wu}^\top & \boldsymbol{\alpha}_{zu|a}^\top\boldsymbol{\Sigma}_{u|a}\boldsymbol{\alpha}_{yu} & (\boldsymbol{\alpha}_{zu|a}^\top\boldsymbol{\Sigma}_{u|a}\boldsymbol{\alpha}_{zu|a} + \Sigma_z) \end{bmatrix} \begin{bmatrix} (\mathbf{I}-\boldsymbol{\Gamma})^{-1} & 0 & 0 \\ 0 & 1 & \alpha_{yz} \\ 0 & 0 & 1 \end{bmatrix}$$

$$= \begin{bmatrix} (\mathbf{I}-\boldsymbol{\Gamma})^{-1}(\boldsymbol{\alpha}_{wu}\boldsymbol{\Sigma}_{u|a}\boldsymbol{\alpha}_{wu}^\top + \boldsymbol{\Sigma}_w)(\mathbf{I}-\boldsymbol{\Gamma})^{-1} & ... & ... \\ \boldsymbol{\alpha}_{yu}^\top\boldsymbol{\Sigma}_{u|a}\boldsymbol{\alpha}_{wu}^\top(\mathbf{I}-\boldsymbol{\Gamma})^{-1} + \boldsymbol{\alpha}_{zu|a}^\top\boldsymbol{\Sigma}_{u|a}\boldsymbol{\alpha}_{wu}^\top(\mathbf{I}-\boldsymbol{\Gamma})^{-1}\alpha_{yz} & (\boldsymbol{\alpha}_{yu}^\top\boldsymbol{\Sigma}_{u|a}\boldsymbol{\alpha}_{yu} + \sigma_y^2) + (\boldsymbol{\alpha}_{zu|a}^\top\boldsymbol{\Sigma}_{u|a}\boldsymbol{\alpha}_{yu})\alpha_{yz} & ... \\ (\boldsymbol{\alpha}_{zu|a}^\top\boldsymbol{\Sigma}_{u|a}\boldsymbol{\alpha}_{wu}^\top)(\mathbf{I}-\boldsymbol{\Gamma})^{-1} & \boldsymbol{\alpha}_{zu|a}^\top\boldsymbol{\Sigma}_{u|a}\boldsymbol{\alpha}_{yu} & ... + \Sigma_z \end{bmatrix}$$

When $\boldsymbol{\Gamma}$ is a zero matrix, we derive Equation (5) and (6). When Γ is not zero matrix but known, the theorem still holds.

Derivation of Equation (9) and (10) for continuous treatment: The equations in (1)-(4) in the main text can be formatted as below, and '0' could be a scalar or vector.

$$\begin{bmatrix} \mathbf{W}_{ij} \\ A_{ij} \\ Y_{ij} \\ Z_{ij} \end{bmatrix} = \begin{bmatrix} \boldsymbol{\Gamma} & 0 & 0 & 0 \\ 0 & 0 & 0 & \alpha_{az} \\ 0 & \theta & 0 & \alpha_{yz} \\ 0 & 0 & 0 & 0 \end{bmatrix} \begin{bmatrix} \mathbf{W}_{ij} \\ A_{ij} \\ Y_{ij} \\ Z_{ij} \end{bmatrix} + \begin{bmatrix} \boldsymbol{\alpha}_{wu} \\ \boldsymbol{\alpha}_{au}^\top \\ \boldsymbol{\alpha}_{yu}^\top \\ 0 \end{bmatrix} \mathbf{U}_{ij} + \begin{bmatrix} \boldsymbol{\epsilon}_w \\ \epsilon_a \\ \epsilon_y \\ \epsilon_z \end{bmatrix}$$

where $\boldsymbol{\Gamma}$ is the auto-correlations between proxies, with zero on diagonal.

Let $\mathbf{B} = \begin{bmatrix} \boldsymbol{\Gamma} & 0 & 0 & 0 \\ 0 & 0 & 0 & \alpha_{az} \\ 0 & \theta & 0 & \alpha_{yz} \\ 0 & 0 & 0 & 0 \end{bmatrix}$, then $(I-\mathbf{B})^{-1} = \begin{bmatrix} \mathbf{I}-\boldsymbol{\Gamma} & 0 & 0 & 0 \\ 0 & 1 & 0 & -\alpha_{az} \\ 0 & -\theta & 1 & -\alpha_{yz} \\ 0 & 0 & 0 & 1 \end{bmatrix}^{-1} = \begin{bmatrix} (I-\boldsymbol{\Gamma})^{-1} & 0 & 0 & 0 \\ 0 & 1 & 0 & \alpha_{az} \\ 0 & \theta & 1 & \alpha_{az}\theta + \alpha_{yz} \\ 0 & 0 & 0 & 1 \end{bmatrix}$

$$\text{Cov}\begin{bmatrix}\mathbf{W}_{ij}\\ A_{ij}\\ Y_{ij}\\ Z_{ij}\end{bmatrix} = \begin{bmatrix}(\mathbf{I}-\mathbf{\Gamma})^{-1} & 0 & 0 & 0\\ 0 & 1 & 0 & \alpha_{az}\\ 0 & \theta & 1 & \alpha_{az}\theta+\alpha_{yz}\\ 0 & 0 & 0 & 1\end{bmatrix}$$

$$\begin{bmatrix}(\boldsymbol{\alpha}_{wu}\mathbf{\Sigma}_u\boldsymbol{\alpha}_{wu}^\top+\mathbf{\Sigma}_w) & \boldsymbol{\alpha}_{wu}\mathbf{\Sigma}_u\boldsymbol{\alpha}_{au} & \boldsymbol{\alpha}_{wu}\mathbf{\Sigma}_u\boldsymbol{\alpha}_{yu} & 0\\ \boldsymbol{\alpha}_{au}^\top\mathbf{\Sigma}_u\boldsymbol{\alpha}_{wu}^\top & (\boldsymbol{\alpha}_{au}^\top\mathbf{\Sigma}_u\boldsymbol{\alpha}_{au}+\sigma_a^2) & \boldsymbol{\alpha}_{au}^\top\mathbf{\Sigma}_u\boldsymbol{\alpha}_{yu} & 0\\ \boldsymbol{\alpha}_{yu}^\top\mathbf{\Sigma}_u\boldsymbol{\alpha}_{wu}^\top & \boldsymbol{\alpha}_{yu}^\top\mathbf{\Sigma}_u\boldsymbol{\alpha}_{au} & (\boldsymbol{\alpha}_{yu}^\top\mathbf{\Sigma}_u\boldsymbol{\alpha}_{yu}+\sigma_y^2) & 0\\ 0 & 0 & 0 & \Sigma_z\end{bmatrix}\begin{bmatrix}(\mathbf{I}-\mathbf{\Gamma})^{-1} & 0 & 0 & 0\\ 0 & 1 & \theta & 0\\ 0 & 0 & 1 & 0\\ 0 & \alpha_{az} & \alpha_{az}\theta+\alpha_{yz} & 1\end{bmatrix}$$

$$= \begin{bmatrix}(\mathbf{I}-\mathbf{\Gamma})^{-1}(\boldsymbol{\alpha}_{wu}\mathbf{\Sigma}_u\boldsymbol{\alpha}_{wu}^\top+\mathbf{\Sigma}_w)(\mathbf{I}-\mathbf{\Gamma})^{-1} & ... & ... & ...\\ \boldsymbol{\alpha}_{au}^\top\mathbf{\Sigma}_u\boldsymbol{\alpha}_{wu}^\top(\mathbf{I}-\mathbf{\Gamma})^{-1} & (\boldsymbol{\alpha}_{au}^\top\mathbf{\Sigma}_u\boldsymbol{\alpha}_{au}+\sigma_a^2)+\alpha_{az}\Sigma_z\alpha_{az} & ... & ...\\ (\theta\boldsymbol{\alpha}_{au}^\top\mathbf{\Sigma}_u\boldsymbol{\alpha}_{wu}^\top+\boldsymbol{\alpha}_{yu}^\top\mathbf{\Sigma}_u\boldsymbol{\alpha}_{wu}^\top)(\mathbf{I}-\mathbf{\Gamma})^{-1} & \theta(\boldsymbol{\alpha}_{au}^\top\mathbf{\Sigma}_u\boldsymbol{\alpha}_{au}+\sigma_a^2)+\boldsymbol{\alpha}_{yu}^\top\mathbf{\Sigma}_u\boldsymbol{\alpha}_{au}+(\alpha_{az}\theta+\alpha_{yz})\Sigma_z\alpha_{az} & ...+\sigma_y^2 & ...\\ 0 & \Sigma_z\alpha_{az} & \Sigma_z(\alpha_{az}\beta+\alpha_{yz}) & \Sigma_z\end{bmatrix}$$

When $\mathbf{\Gamma}$ is a zero matrix, we derive Equation (9) and (10). When $\mathbf{\Gamma}$ is not zero matrix but known, the theorem still holds.

Web Appendix C: Computational details

Latent factor model: We use Metropolis-Hastings Sampling to update the latent process $\mathbf{U}_{ij}$ and λ_{il} city-by-city. We use prior $\mathbf{U}_{ij} \overset{iid}{\sim} \mathcal{N}(0, \text{diag}(\sigma_{u_1}^2, ..., \sigma_{u_q}^2))$ for any i and j, $\lambda_{il} \overset{iid}{\sim} \mathcal{N}(0, \sigma_z^2)$ for any i and l, and $\sigma_{u_1}^2, ..., \sigma_{u_q}^2, \sigma_z^2 \overset{iid}{\sim} \text{InvGamma}(0.01, 0.01)$. In practice, we set $\alpha_{w_1u} = 1$ to scale U_{ij1} by W_{ij1}.

Normally distributed proxy: We use Gibbs sampling for this standard regression model with parameters $\boldsymbol{\alpha}_w, \boldsymbol{\alpha}_{wu}, \sigma_{w_1}^2, ..., \sigma_{w_p}^2$. We use priors for each element of $\boldsymbol{\alpha}_w, \boldsymbol{\alpha}_{wu}$ to be i.i.d. $\mathcal{N}(0, \tau_w^2)$, and $\tau_w^2, \sigma_{w_1}^2, ..., \sigma_{w_p}^2 \overset{iid}{\sim} \text{InvGamma}(0.01, 0.01)$.

Zero-inflated proxy: The variable percentage of Black population (denoted as W_{ij1} for convenience in this paragraph) has around 5% values being zeros. We transform it by rank-based inverse normal and fit it with a zero-inflated Tobit model. Let w_L denote the censoring boundary (meaning percent of Black population is zero). The evaluated likelihood function in city i is:

$$\mathcal{L}(\cdot; W_{ij1}, \mathbf{U}_{ij}) = \prod^{i}\left(\frac{1}{\sigma_{w_1}}\phi\left(\frac{W_{ij1}-h(\mathbf{U}_{ij})}{\sigma_{w_1}}\right)\right)^{I(W_{ij1})}\prod^{i}\left(1-\Phi\left(\frac{h(\mathbf{U}_{ij})-w_L}{\sigma_{w_1}}\right)\right)^{1-I(W_{ij1})}$$

where $I(w) = \begin{cases}0, & w \le w_L\\ 1, & w > w_L\end{cases}$; ϕ and Φ denote the density function and cumulative density function of the standard normal distribution, respectively; $h(\mathbf{U}_{ij})$ denotes the conditional expectation $h(\mathbf{U}_{ij}) = \alpha_{w_1} + \boldsymbol{\alpha}_{w_1u}\mathbf{U}_{ij}$. We use Metropolis-Hastings sampling to update all parameters with a standard acceptance probability. We use priors $\alpha_{w_1}, \boldsymbol{\alpha}_{w_1u} \overset{iid}{\sim} \mathcal{N}(0, \tau_w^2)$, and $\tau_{w_1}^2, \sigma_{w_1}^2 \sim \text{InvGamma}(0.01, 0.01)$. The candidate of $\sigma_{w_1}^2$ is obtained from a log-normal distribution to ensure positivity.

Treatment model: The treatment model is modeled $\text{logit}(P(A_{ij} = 1|\mathbf{U}_{ij}, Z_{ij})) = \alpha_a + \boldsymbol{\alpha}_{au}\mathbf{U}_{ij} + \alpha_{az}Z_{ij}$. We use Metropolis-Hastings Sampling to update parameters $\alpha_a, \boldsymbol{\alpha}_{au}$. We use

priors for each element of $\alpha_a, \boldsymbol{\alpha}_{au} \overset{iid}{\sim} \mathcal{N}(0, \tau_1^2)$, $\tau_1^2 \sim$ InvGamma$(0.01, 0.01)$. We set $\alpha_{az} = 1$ to scale the values of latent Z_{ij}.

Multiple treatments: If there are multiple treatment categories, we assume a multinomial logistic regression. Let A_{ij} be the treatment category of unit (i, j), taking values in $\{1, \ldots, C\}$. We model with $\Pr(A_{ij} = c \mid \mathbf{U}_{ij}, Z_{ij}) = \frac{\exp\left(\alpha_c + \boldsymbol{\alpha}_{cu}^\top \mathbf{U}_{ij} + \alpha_{cz} Z_{ij}\right)}{\sum_{r=1}^{C} \exp\left(\alpha_c + \boldsymbol{\alpha}_{cu}^\top \mathbf{U}_{ij} + \alpha_{cz} Z_{ij}\right)}, \quad c = 1, \ldots, C$. We fix the last category as the 'reference' without modeling it. We use priors for each element of $\alpha_c, \boldsymbol{\alpha}_{cu} \overset{iid}{\sim} \mathcal{N}(0, \tau_1^2)$, $\tau_1^2 \sim$ InvGamma$(0.01, 0.01)$, and update using Metropolis-Hastings Sampling.

Outcome model: The outcome model is $Y_{ij} | A_{ij}, \mathbf{U}_{ij}, Z_{ij} \sim \mathcal{N}(\alpha_y + \theta A_{ij} + \boldsymbol{\alpha}_{yu} \mathbf{U}_{ij} + \alpha_{yz} Z_{ij}, \sigma_y^2)$. We use Gibbs Sampling to update all involved parameters. We use priors $\alpha_y, \theta, \boldsymbol{\alpha}_{yu} \sim \mathcal{N}(0, \tau_2^2)$, $\alpha_{yz} \sim \mathcal{N}(0, \tau_3^2)$, $\sigma_y^2, \tau_2^2, \tau_3^2 \sim$ InvGamma$(0.01, 0.01)$.

Random effect model: Denote the city-level random effects $\theta_1, \ldots, \theta_M \overset{iid}{\sim} \mathcal{N}(\mu_\theta, \sigma_\theta^2)$. We use priors $\mu_\theta \sim \mathcal{N}(0, 100^2)$, $\sigma_\theta^2 \sim$ InvGamma$(0.01, 0.01)$. This model uses the posterior parameter estimates from the constant effect model as initial values, which significantly speed up convergence.

Other Implementation Details: We fit the models via MCMC and obtained posterior samples of all the unknown parameters. The data and statistical models fit with R 4.2.3 have been made available with the paper. JABS/BUGS is not preferred due to computational efficiency.

Web Appendix D: Additional results for simulation study

To create data with simple grid geometry and predetermined parameters, we generate 490 regions in 10 cities, consisting of 7-by-7 grid regions in each city. The data-generation process is defined by

$$
\begin{aligned}
&U_{ij} \sim \mathcal{N}(0,1), \quad Z_{ij} = \sum_{l=1}^{L_i} \lambda_{il} B_{ijl}, \quad \lambda_{il} \sim \mathcal{N}(0,1), \quad W_{ij1} \sim \mathcal{N}(\alpha_{w_1 u} U_{ij}, \sigma_{w_1}^2), \\
&W_{ij2} \sim \mathcal{N}(\alpha_{w_2 u} U_{ij}, \sigma_{w_2}^2), \quad \widetilde{W}_{ij3} \sim \mathcal{N}(\alpha_{w_3 u} U_{ij}, \sigma_{w_3}^2), \quad W_{ij3} = \max(\widetilde{W}_{ij3}, \text{quantile}_{.05}(\widetilde{W}_3)), \\
&\text{logit}(P(A_{ij} = 1)) = \alpha_a + \alpha_{au} U_{ij} + \alpha_{az} Z_{ij}, \quad Y_{ij} \sim \mathcal{N}(\theta A_{ij} + \alpha_{yu} U_{ij} + \alpha_{yz} Z_{ij}, \sigma_y^2).
\end{aligned}
$$

We consider six cases with correctly specified model in the main paper. The first is a base case with (1) $\theta = 0.2$, $\alpha_{w_1 u} = 2$, $\alpha_{w_2 u} = -0.5$, $\alpha_{w_3 u} = -1$, $\sigma_{w_1}^2 = \sigma_{w_2}^2 = \sigma_{w_3}^2 = 1$, $r = 40\%$, $\alpha_a = -1$, $\alpha_{au} = -1$, $\alpha_{az} = 1$, $\alpha_{yu} = -0.2$, $\alpha_{yz} = -1$, $\sigma_y^2 = 0.25$. The others cases modify the base case as follows: (2) Stronger proxy: $\sigma_{w_1}^2 = \sigma_{w_2}^2 = \sigma_{w_3}^2 = 0.5$, (3) Noisier outcome: $\sigma_y^2 = 1$, and (4) Rougher spatial confounding: $r = 60\%$. (5) Weaker proxy: $\sigma_{w_1}^2 = \sigma_{w_2}^2 = \sigma_{w_3}^2 = 2$, (6) Stronger confounder **U**: $\alpha_{au} = -2, \alpha_{yu} = -2$.

In this appendix, we extended the simulation study by modifying the base case (1) to include scenarios: (7) Misspecified model on $\mathbb{E}(A|U)$ and $\mathbb{E}(Y|U)$, both A and Y depends on W_1, W_2, W_3 directly: $\text{logit}(P(A_{ij} = 1)) = \alpha_a + \alpha_{au}(-W_{ij1} + W_{ij2} + W_{ij3}) + \alpha_{az} Z_{ij}$, $Y_{ij} \sim \mathcal{N}(\theta A_{ij} + \alpha_{yu}(-W_{ij1} + W_{ij2} + W_{ij3}) + 0.2 U_{ij} Z_{ij} + \alpha_{yz} Z_{ij}, \sigma_y^2)$, and U enters through an interaction with Z. (8) Misspecified model on $\mathbb{E}(Y|U, Z)$: generate data with $Y_{ij} \sim \mathcal{N}(\theta A_{ij} + \alpha_{yu} U_{ij} + \alpha_{yz} Z_{ij} + 0.2 U_{ij} Z_{ij}, \sigma_y^2)$, (9) Misspecified model on $\mathbb{E}(Y|U)$: generate data with $Y_{ij} \sim$

$\mathcal{N}(\theta A_{ij} + \alpha_{yu} U_{ij}^2 + \alpha_{yz} Z_{ij}, \sigma_y^2)$, (10) Misspecified model on $\mathbb{E}(Y|U)$: generate data with $Y_{ij} \sim \mathcal{N}(\theta A_{ij} + \alpha_{yu} log(|U_{ij}|) + \alpha_{yz} Z_{ij}, \sigma_y^2)$. In all cases, W_{ij3} is generated to have the lowest 5% percent values as zero, to model the zero-inflated percentage of Black Population in the real data. (11) Misspecified model on distribution of U: generate data with $U_{ij} \sim \mathcal{N}(\sum_{l=1}^{L_i} \kappa_{il} B_{ijl}, 1)$, where $\kappa_{il} \sim \mathcal{N}(0, 1)$. The results are shown in Web Table S2.

In setting (8), the outcome depends on an interaction between U and Z, yet our model still performs well. In setting (7), both treatment and outcome depend directly on the proxies in a complex way rather than through the latent factor. As a result, our coverage is poor in this case, while the outcome-regression-with-proxy approach performs relatively better. In setting (9), the outcome depends on the square of U, and in setting (10) on a non-monotone transformation $\log(|U|)$; in both cases, our coverage deteriorates. These weaknesses arise because the structural causal model is violated.

In our substantive application, while Assumption 4 cannot be directly verified, the observed proxies for U (unemployment, house rent, racial composition) are linearly associated with each other (Web Figure S6), and both NO_2 and $PM_{2.5}$ show relationships with these proxies that do not appear to be grossly violated (Web Figure S7). These patterns provide indirect evidence for representing socio-economic disadvantage as a latent factor with a monotone, approximately linear effect on air pollution. We therefore view settings (7), (9), and (10) as stress tests rather than realistic scenarios for our application.

Case (11) allows U to have a spatial component, relaxing the assumption that the latent factor U is i.i.d. and thereby inducing spatial correlation in the proxies W. This design more closely reflects the empirical redlining data, where variables such as unemployment, rent, and racial composition display strong spatial structure. Our latent adjustment method continues to achieve good coverage in this setting. The robustness arises because U and the spatial process Z play symmetric roles in the structural model, allowing the spatial component of U to be ‘absorbed’ by the latent spatial factor Z.

Web Table S2: Additional simulation results. In all cases, the true spline ratio is 40%. The columns display the average absolute bias (A.B.) with standard deviation, mean square error (MSE) with standard deviation, coverage probability (%, C.P.), Watanabe-Akaike Information Criterion (WAIC), and the selected spline ratio (S.R.).

Case	Method	A.B.	MSE	C.P.	WAIC	S.R.
(7)	Latent Adjustment	0.413 (0.205)	0.212 (0.212)	13	-407	44
	Outcome Regr with Proxy	0.024 (0.114)	0.013 (0.020)	88	1045	45
	No Adjustment	3.595 (0.127)	12.938 (0.911)	0	1938	5
(8)	Latent Adjustment	0.013 (0.098)	0.010 (0.014)	96	1082	55
	Outcome Regr with Proxy	0.234 (0.081)	0.061 (0.039)	16	1118	46
	No Adjustment	1.192 (0.096)	1.430 (0.229)	0	1467	41
(9)	Latent Adjustment	0.635 (0.343)	0.520 (0.688)	28	1853	21
	Outcome Regr with Proxy	0.483 (0.236)	0.288 (0.242)	30	1856	17
	No Adjustment	0.443 (0.182)	0.229 (0.163)	22	1854	18
(10)	Latent Adjustment	0.435 (0.223)	0.238 (0.229)	38	1659	33
	Outcome Regr with Proxy	0.370 (0.203)	0.177 (0.177)	44	1666	30
	No Adjustment	0.284 (0.138)	0.099 (0.098)	47	1659	34
(11)	Latent Adjustment	0.026 (0.107)	0.012 (0.019)	91	1090	54
	Outcome Regr with Proxy	0.266 (0.089)	0.079 (0.048)	15	1122	44
	No Adjustment	1.320 (0.106)	1.766 (0.277)	0	1497	42

Web Appendix E: Additional results for redlining policy analysis

We considered a cumulative 40-year period and the exposed populations in our dataset. We used a historically appropriate baseline mortality rate of 900 deaths per 100,000 persons per year (Hoyert, 2012). We drew hazard ratios uniformly from published 95% confidence intervals: $\mathrm{HR}_{10} = 1.02$ (95% CI: 1.01-1.04) per 10 $\mu g/m^3$ per year for NO2, and $\mathrm{HR}_{10} = 1.08$ (95% CI: 1.06–1.09) per 10 $\mu g/m^3$ per year for PM2.5 (WHO, 2021). We convert NO_2 concentrations using 1 ppb $\approx 1.914\,\mu g/m^3$. For each pollutant, let Δ denote the redlining-attributed concentration difference (in $\mu g/m^3$). To propagate uncertainty, we conducted Monte Carlo simulations (10,000 draws), sampling Δ from posterior distribution. For each draw, the excess mortality fraction is

$$\mathrm{HR}_{\Delta} = \mathrm{HR}_{10}^{(\Delta/10)}.$$

Then the mortality increase (fraction) is

$$\mathrm{MI} = \mathrm{HR}_{\Delta} - 1 = \mathrm{HR}_{10}^{(\Delta/10)} - 1,$$

and cumulative excess deaths are

$$\text{Excess deaths} = (\text{baseline death rate}) \times (\text{population}) \times (\text{years}) \times \mathrm{MI}.$$

Final estimates are reported as the Monte Carlo mean and 95% simulation interval, shown in Web Table S5. Results indicate that, although exposure differences are small in absolute terms, when scaled to affected populations and decades of exposure they imply non-trivial public-health consequences.

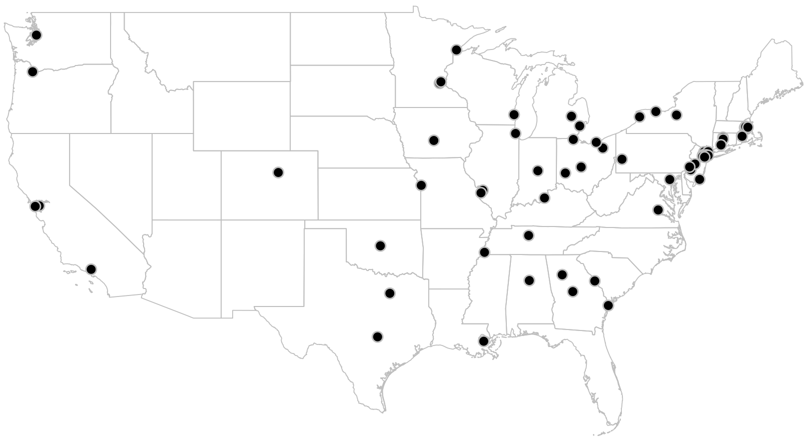

Web Figure S2: Map showing the distribution of 69 cities across 27 states included in our study.

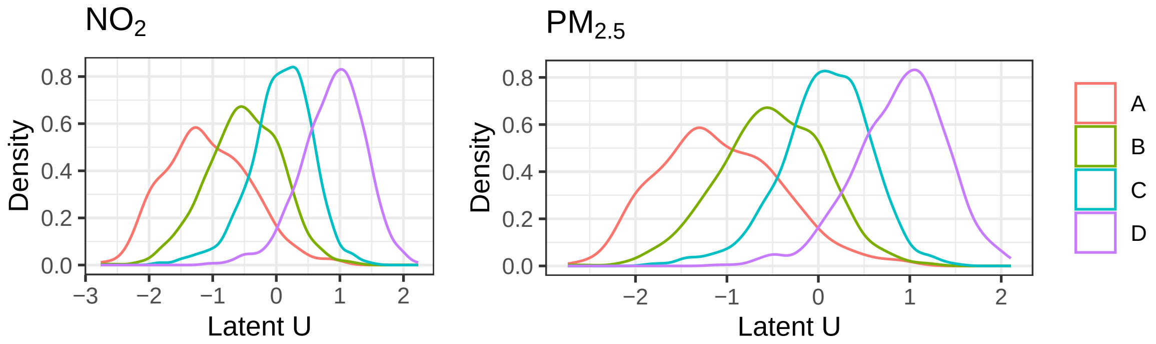

Web Figure S3: Overlap of latent U_{ij} in the redlining data analysis with $r = 60\%$ for NO_2 (left) and $PM_{2.5}$ (right). These density plots confirm that the overlap assumption is met, ensuring that there is sufficient overlap between the treatment and control groups for valid causal inference.

Web Table S3: Positivity of treatment assignment in the redlining data analysis with $r = 60\%$. The values indicate the probability of being redlined for a given value of U_{ij} at specific quantiles of the U_{ij} distribution, controlling $Z_{ij} = 0$. This table confirms that the positivity assumption is moderately met.

Outcome	Grades	Q0	Q2.5	Q25	Q50	Q75	Q97.5	Q100
NO_2	A	0.902	0.566	0.118	0.043	0.016	0.003	0.001
NO_2	B	0.097	0.392	0.436	0.236	0.108	0.021	0.005
NO_2	C	0.001	0.042	0.428	0.605	0.511	0.115	0.018
NO_2	D	0	0	0.019	0.116	0.364	0.861	0.976
$PM_{2.5}$	A	0.897	0.558	0.116	0.043	0.016	0.003	0.001
$PM_{2.5}$	B	0.101	0.397	0.433	0.236	0.106	0.021	0.007
$PM_{2.5}$	C	0.002	0.044	0.431	0.604	0.507	0.118	0.029
$PM_{2.5}$	D	0	0	0.02	0.117	0.371	0.858	0.963

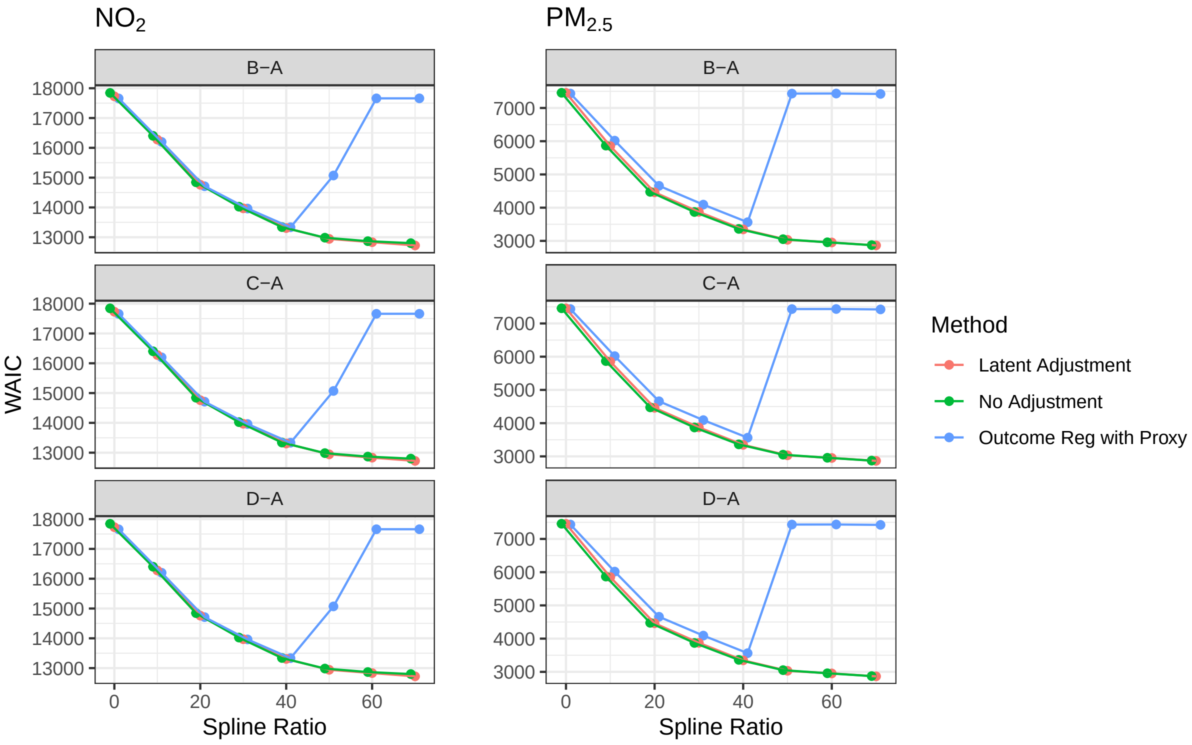

Web Figure S4: WAIC of outcome models for redlining real data analysis. The left panel for NO_2 and right for $PM_{2.5}$. The x-axis is the spline ratio r and y-axis is WAIC. These plots demonstrate that the Latent Adjustment achieves the lowest WAIC values compared to the No Adjustment and Outcome Regression with Proxy methods for a wide ranges of spline ratio.

Web Table S4: Posterior estimates of parameters ($\alpha_{w_1u}, \alpha_{w_2u}, \alpha_{w_3u}, \alpha_{au}, \alpha_{yu}$) representing the relationship between the latent U_{ij} and other variables in the redlining data analysis when $r = 60\%$. The values are comparable within columns but not across rows. The absolute values contains limited information, since the data are transformed, scaled, and centered. This table illustrates that, whether the outcome is $PM_{2.5}$ or NO_2, we consistently identify a latent factor U_{ij} representing socio-economic status (SES) as expected: higher values of U_{ij} correspond to lower SES and are numerically associated with higher unemployment rates, lower housing rent, a higher percentage of Black population, increased probability of being redlined, and elevated air pollution levels.

Outcome	Unemployment Rate	House Rent	Percent of Black	Prob Graded B	Prob Graded C	Prob Graded D	Outcome
NO_2	1.00	-0.97	0.20	1.90	1.89	2.54	0.61
$PM_{2.5}$	1.00	-0.96	0.20	1.90	1.87	2.49	0.05

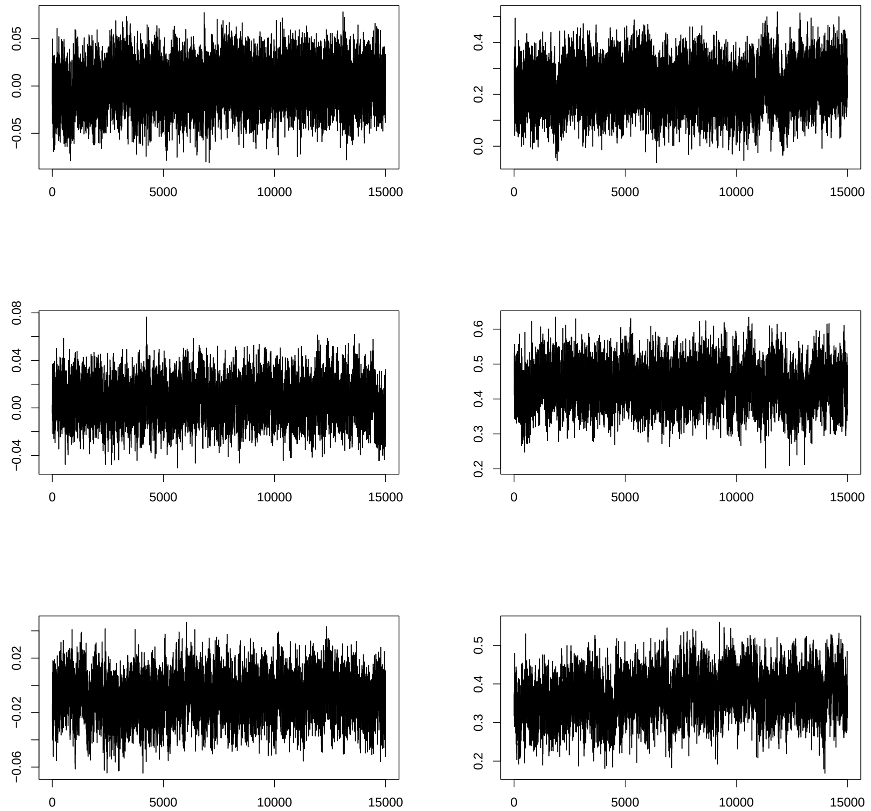

Web Figure S5: Example trace plot with 150,000 iterations after burn-in for relining data analysis. $PM_{2.5}$ is shown on the left, NO_2 on the right. Form top to bottom, the values of r are 10%, 40%, 70%. he trace plots indicate stable convergence of multiple models.

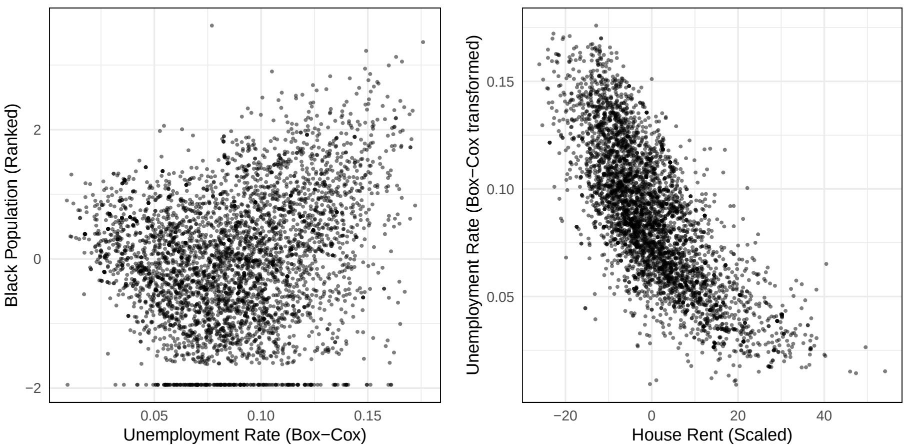

Web Figure S6: Pairwise associations among proxies for latent socio-economic status.

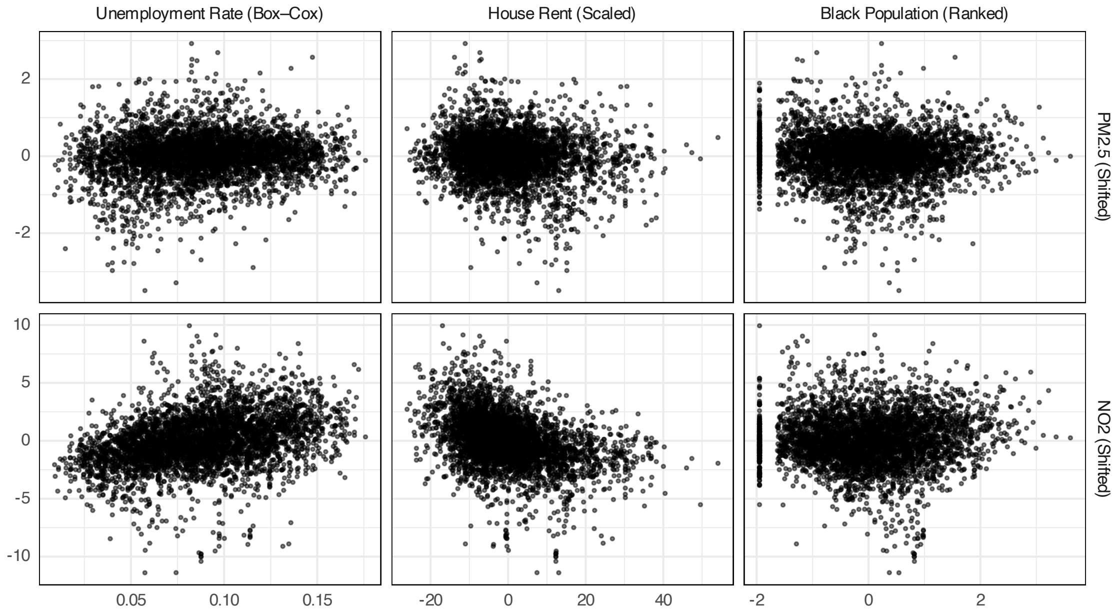

Web Figure S7: Associations between air pollution outcomes and socio-economic proxies.

	Grade B versus A	Grade C versus A	Grade D versus A
NO_2	3,435 (1,405 to 5,973)	14,393 (6,046 to 23,934)	14,869 (6,167 to 25,386)
$PM_{2.5}$	767 (319 to 1,254)	1,622 (387 to 2,936)	1,046 (-524 to 2,653)
Total	4,202 (1,723 to 7,226)	16,015 (6,433 to 26,870)	15,915 (5,642 to 28,039)

Web Table S5: Cumulative excess deaths over 40 years.

References

Anderson, T. and Rubin, H. (1956). Statistical Inference in Factor Analysis. In *Proceedings of the Berkeley Symposium on Mathematical Statistics and Probability*, page 111. University of California Press.

Hoyert, D. L. (2012). *75 Years of Mortality in the United States, 1935-2010*. Number 76. U.S. Department of Health and Human Services, Centers for Disease Control and Prevention, National Center for Health Statistics.

Kang, S., Franks, A., and Antonelli, J. (2025). Sensitivity Analysis With Multiple Treatments and Multiple Outcomes With Applications to Air Pollution Mixtures. *arXiv:2311.12252v3* .

Miao, W., Hu, W., Ogburn, E. L., and Zhou, X.-H. (2023). Identifying Effects of Multiple Treatments in the Presence of Unmeasured Confounding. *Journal of the American Statistical Association* **118,** 1953–1967.

WHO (2021). *WHO Global Air Quality Guidelines*. World Health Organization.
